# Agonistic Activation of Cytosolic DNA Sensing Receptors in Woodchuck Hepatocyte Cultures and Liver for Inducing Antiviral Effects

**DOI:** 10.3389/fimmu.2021.745802

**Published:** 2021-10-04

**Authors:** Manasa Suresh, Bin Li, Xu Huang, Kyle E. Korolowicz, Marta G. Murreddu, Severin O. Gudima, Stephan Menne

**Affiliations:** ^1^ Department of Microbiology & Immunology, Georgetown University Medical Center, Washington, DC, United States; ^2^ Department of Microbiology, Molecular Genetics & Immunology, University of Kansas Medical Center, Kansas City, KS, United States

**Keywords:** pattern recognition receptors, hepatitis B virus, viral DNA sensing receptors, innate immune response, woodchuck, chronic hepatitis B

## Abstract

Immune modulation for the treatment of chronic hepatitis B (CHB) has gained more traction in recent years, with an increasing number of compounds designed for targeting different host pattern recognition receptors (PRRs). These agonistic molecules activate the receptor signaling pathway and trigger an innate immune response that will eventually shape the adaptive immunity for control of chronic infection with hepatitis B virus (HBV). While definitive recognition of HBV nucleic acids by PRRs during viral infection still needs to be elucidated, several viral RNA sensing receptors, including toll-like receptors 7/8/9 and retinoic acid inducible gene-I-like receptors, are explored preclinically and clinically as possible anti-HBV targets. The antiviral potential of viral DNA sensing receptors is less investigated. In the present study, treatment of primary woodchuck hepatocytes generated from animals with CHB with HSV-60 or poly(dA:dT) agonists resulted in increased expression of interferon-gamma inducible protein 16 (IFI16) or Z-DNA-binding protein 1 (ZBP1/DAI) and absent in melanoma 2 (AIM2) receptors and their respective adaptor molecules and effector cytokines. Cytosolic DNA sensing receptor pathway activation correlated with a decline in woodchuck hepatitis virus (WHV) replication and secretion in these cells. Combination treatment with HSV-60 and poly(dA:dT) achieved a superior antiviral effect over monotreatment with either agonist that was associated with an increased expression of effector cytokines. The antiviral effect, however, could not be enhanced further by providing additional type-I interferons (IFNs) exogenously, indicating a saturated level of effector cytokines produced by these receptors following agonism. In WHV-uninfected woodchucks, a single poly(dA:dT) dose administered *via* liver-targeted delivery was well-tolerated and induced the intrahepatic expression of ZBP1/DAI and AIM2 receptors and their effector cytokines, IFN-β and interleukins 1β and 18. Receptor agonism also resulted in increased IFN-γ secretion of peripheral blood cells. Altogether, the effect on WHV replication and secretion following *in vitro* activation of IFI16, ZBP1/DAI, and AIM2 receptor pathways suggested an antiviral benefit of targeting more than one cytosolic DNA receptor. In addition, the *in vivo* activation of ZBP1/DAI and AIM2 receptor pathways in liver indicated the feasibility of the agonist delivery approach for future evaluation of therapeutic efficacy against HBV in woodchucks with CHB.

## Introduction

Hepatitis B virus (HBV) is the leading cause of the global hepatitis burden, with an estimated 296 million chronic carriers and an annual virus-related death toll of 820,000 (1). HBV infection acquired early in life by mother-to-child transfer or as an infant will progress to chronicity in more than 95% of cases and carriers have a high risk of developing serious liver disorders, including chronic hepatitis B (CHB), cirrhosis, and liver cancer or hepatocellular carcinoma (HCC) (2). HBV is a member of the *hepadnaviridae* family and replicates in hepatocytes by forming a reservoir of genomic DNA in the nucleus. This so-called covalently-closed circular (ccc) DNA molecule serves as the template for replication intermediate products and viral proteins. The complex HBV life cycle and the continuous replenishment of the cccDNA pool in infected hepatocytes are significant challenges for anti-HBV drug development. The current treatment options for CHB are suboptimal and rarely lead to a cure of the infection. The available nucleos(t)ide analogs are well-tolerated, but do not target the cccDNA directly, and therefore treatment cessation leads to viral rebound making their life-long administration necessary (3, 4). While systemic pegylated interferon (IFN) alpha therapy can directly target the cccDNA, it is effective only in a subset of treated patients and frequently associated with severe side effects (3, 4). In recent years, anti-HBV drug discovery has focused on direct acting antivirals and immunomodulators for targeting different steps of the HBV life cycle or for restoring the dysfunctional antiviral immune response present in patients with CHB, respectively (5).

Modulating the immune response against CHB has been explored by using several approaches, including agonistic activation of innate immune receptors, treatment with checkpoint inhibitors, adoptive transfer of T-cells, and administration of therapeutic vaccines (6–9). These approaches have demonstrated promising results in preclinical studies as they induced HBV-specific immune responses, and thus were able to overcome the impaired immunity associated with CHB. However, some of the challenges that need to be addressed in regard to immunomodulation are the systemic toxicity due to non-specific delivery of compounds and the risk of uncontrollable liver damage (i.e., acute liver failure) when breaking immune tolerance (10, 11). Furthermore, the lack of small, immunocompetent laboratory animal models for testing experimental therapeutics against HBV is an additional hurdle. The infection of the Eastern woodchuck (*Marmota monax*) with woodchuck hepatitis virus (WHV) resembles the vertical HBV transmission in humans, including immunopathogenesis and liver disease progression (12, 13). Like HBV, the outcome of WHV infection is age dependent, with 60-75% of WHV-infected neonatal woodchucks progressing to chronicity, while less than 5% of WHV-infected adult woodchucks develop CHB (14). This immunocompetent animal model is, therefore, intensively applied in the evaluation of the safety and therapeutic efficacy of novel anti-HBV drugs *in vitro* and *in vivo*.

The ability of the host immune system to fight viral infections is dependent on germline-encoded pattern recognition receptors (PRRs) that recognize unique motifs called pathogen-associated molecular patterns (PAMPs), such as viral RNA, DNA, and proteins (15). The subcellular localization of these receptors in both immune and non-immune cells is either extracellular on plasma membranes or intracellular on endosomal membranes or within the cytoplasm, but sometimes also in the nucleus. The widely studied families of PRRs include the retinoic acid-inducible gene-I like receptors (RLRs), the nucleotide-binding oligomerization domain containing protein 2 (NOD2) like receptors (NLRs), toll-like receptors (TLRs), cytosolic DNA sensors (CDSs), and inflammasomes. Stimulation of these receptors triggers their downstream signaling pathway involving adaptor molecules, transcription factors, and type-I IFNs or pro-inflammatory cytokines, and leads to an antiviral immune response (16, 17). Unlike many other viruses, HBV fails to induce a type-I IFN response immediately after infection and hence is referred to a stealth-like virus (18). While *in vitro* studies have demonstrated that HBV proteins can interfere with the receptor pathway activation (19–21), other studies have argued that the virus does not actively inhibit the function of PRRs, and that these receptors can be agonistically stimulated in the setting of CHB (22).

The safety and antiviral efficacy against HBV associated with the stimulation of mainly viral RNA sensing receptors by small agonist molecules have been investigated in preclinical studies and several compounds were subsequently tested in patients with CHB (7, 10). Within the TLR family, GS-9620 was the first-in-class TLR7 agonist that significantly suppressed viral replication in HBV-infected chimpanzees (23) and in WHV-infected woodchucks (24) as a single agent. However, this compound failed to mediate therapeutic efficacy in patients at tolerated doses (25). Monotreatment with the TLR8 agonist GS-9688 also mediated sustained antiviral effects in a subset of woodchucks (26). Other TLR7 and 9 agonists tested in the woodchuck model produced superior antiviral effects only when administered in combination with the nucleoside analog entecavir (ETV) (27–29). Apart from these TLR agonists, SB9200, a RIG-I stimulator with both immunomodulatory and direct antiviral properties (30, 31) mediated pronounced therapeutic efficacy in woodchucks when the immune response was activated by this agonist before additional viral suppression with ETV (32). Except for TLR9, the antiviral potential of viral DNA sensing receptors against CHB, especially that of CDSs and inflammasomes, is less explored. The reason for this lack in knowledge is that many of these DNA receptors, such as interferon-gamma inducible protein 16 (IFI16), Z-DNA-binding protein 1 or DNA-dependent activator of interferon regulatory factors (ZBP1/DAI), cyclic GMP-AMP synthase (cGAS), and absent in melanoma 2 (AIM2) inflammasome, were characterized more recently for their structural properties, downstream signaling pathways, and role in infectious diseases and autoimmune disorders (33). IFI16 and ZBP1/DAI receptors recognize viral double-stranded (ds) DNA and mediate the production of type-I IFNs by recruiting the stimulator of interferon genes (STING) as adaptor molecule or by activating the TANK-binding kinase 1 (TBK1) and interferon regulatory factor 3 (IRF3) pathway, respectively (34, 35). A recent *in vitro* study further demonstrated that nucleus resident IFI16 can recognize HBV cccDNA and that receptor overexpression affects HBV replication (36). The AIM2 inflammasome recognizes viral dsDNA and initiates the formation of a multi-protein complex consisting of the receptor, the adaptor protein apoptosis-associated speck-like protein containing a CARD domain (ASC), and caspase-1 for the production of the pro-inflammatory cytokines, interleukins (IL) 1β and 18 (35). The molecular characterization of IFI16 and AIM2 receptors in woodchucks and their involvement in the control of WHV infection were described recently (37, 38). The present study reports the effects on WHV replication and secretion that are mediated by mono and combination treatment with the agonists HSV-60 and poly(dA:dT) for targeting the IFI16, ZBP1/DAI, and AIM2 receptors in primary hepatocytes generated from woodchucks with CHB. In addition, the safety and activity of a low and high dose of the poly(dA:dT) agonist for the induction of ZBP1/DAI and AIM2 receptors in the liver of WHV-uninfected woodchucks are described.

## Materials and Methods

### Ethics Statement

The animal protocol # 2019-0064 entitled “Pharmacodynamic and tolerability study of innate immune receptor agonists in woodchucks” and all procedures involving woodchucks were approved by the Institutional Animal Care and Use Committee of Georgetown University on October 22, 2019 and followed the National Institutes of Health guidelines for the Care and Use of Laboratory Animals. Woodchuck were anesthetized by inhalation of isoflurane (3-5%) and by intramuscular injection of ketamine (50 mg/kg) and xylazine (5 mg/kg) for blood collection or percutaneous liver biopsy, respectively. Prior to euthanasia, woodchucks were anesthetized as described above and euthanized by an overdose of pentobarbital (80-100 mg/kg) administered by intracardiac injection, followed by bilateral intercostal thoracotomy.

### Woodchuck Hepatocyte Isolation and PRR Stimulation

Primary woodchuck hepatocytes (PWHs) were generated from animals with CHB using the collagenase perfusion method and maintained for four days before treatment initiation (39). The agonists HSV-60 (Invivogen, San Diego, CA) and poly(dA:dT) (Invivogen) targeting the IFI16 or ZBP1/DAI and AIM2 receptors, respectively, were administered to PWHs at doses of 2.0 or 1.0 µg/mL, respectively, at T0 and again after 48 hours using Lipofectamine 3000 (Thermo Fisher Scientific, Waltham, MA). Lipofectamine-containing medium without agonists served as the untreated control. Cell supernatant and hepatocytes were collected every 24 hours over a 96-hour time course. Isolation of peripheral blood mononuclear cells (PBMCs) and treatment with the TLR7 agonist GS-9620 or IFN-α are described in the **Supplementary Material**.

### *In Vitro* Receptor Pathway Activation and WHV Replication

The expression of receptors, downstream adaptor molecules, and effector cytokines (**Supplementary Table 1**) in PWHs and woodchuck hepatoma cells was determined by real-time PCR and woodchuck-specific primers and probes (**Supplementary Table 2**) as described previously (38, 40). WHV relaxed circular- (rc-) DNA was isolated from cell supernatant, while WHV pre-genomic (pg) RNA and WHV cccDNA were isolated from PWHs and quantified using real-time PCR as described previously (39, 41) and in the Supplementary Material.

### Woodchuck Study Design

The two WHV-uninfected adult woodchucks, M8001 and F8003, were confirmed negative for serum WHV DNA, WHV surface antigen (WHsAg), and antibodies to WHsAg (anti-WHs antibodies) using assays described previously (29). Both animals were intravenously injected with a single dose of poly(dA:dT) (Invivogen) mixed in *in vivo*-jetPEI-Gal transfection reagent (PolyPlus Transfection, Illkirch, France), as described in the Supplementary Material. Woodchuck M8001 received a low dose (125 µg/kg), while animal F8003 received a high dose (375 µg/kg) of poly(dA:dT). Blood samples were collected into PAXGene tubes (Qiagen, Redwood City, CA) at pre-treatment, and then at 24-hours post-treatment for analyzing gene expression. Ultrasound-guided, percutaneous liver biopsies were also obtained at pre-treatment and at 24-hours post-treatment, placed immediately in liquid nitrogen, and stored at 80°C for subsequent gene expression analysis. Body weights, body temperatures, hematology, clinical chemistry, and liver histology of both woodchucks were frequently determined.

### Whole Blood Assay

The effective delivery of poly(dA:dT) with the PolyPlus family of transfection reagents was first tested *in vitro* by using the jetOPTIMUS transfection reagent, and before the subsequent *in vivo* administration of the agonist to woodchucks with the *in vivo*-jetPEI-Gal transfection reagent. The jetOPTIMUS transfection reagent is specifically designed for *in vitro* transfection of hard-to-transfect cells, including primary cells/blood cells (42). Whole blood from animals M8001 and F8003 was drawn into hirudin-coated blood collection tubes (Sarstedt, Numbrecht, Germany). A total of 185 µL of blood was then transferred into the wells of a 96-well round bottom plate (Corning, Tewksbury, MA) and incubated for 6 hours with 15 µL of jetOPTIMUS transfection reagent containing poly(dA:dT). As an untreated control, blood samples were incubated with transfection reagent only. Thereafter, blood was collected from 5 wells of treated or untreated control samples and combined, cells were lysed with RLT buffer (Qiagen) containing 1% β-mercaptoethanol, and total RNA was isolated using the QIAamp RNA Blood Mini kit (Qiagen). The mRNA was subsequently converted to complementary (c) DNA and the expression of receptors, adaptor molecules, and effector cytokines was determined by real time PCR assay, as described recently (38, 40). A fold-change of ≥2.1 from the untreated control was considered a positive result for increased gene expression after *in vitro* treatment with poly(dA:dT).

### ELISpot Assay

PBMCs were isolated from woodchucks M8001 and F8003 by Ficoll-Paque density gradient centrifugation, as described in the Supplementary Material. The ELISpot assay for measuring IFN-γ secretion by blood cells was performed with a commercially available kit (Mabtech, Stockholm, Sweden) by following the manufacturer’s protocol. In brief, each well of a polyvinylidene fluoride plate was pretreated with 50 μL of 70% ethanol for 2 minutes and washed with sterile water before coating with 100 μL/well of IFN-γ antibody (15 μg/mL). Following overnight incubation at 4°C, excess antibody was removed by washing five times with phosphate-buffered saline (PBS). A total of 200 μL of AIM-V cell culture medium (Thermo Fisher Scientific) was then added to each well and incubated for 30 minutes. Thereafter, PBMCs ranging from 50,000 to 125,000 cells/well were added and the plate was incubated at 37°C and 5% CO_2_ for 12-48 hours. Following the incubation, cells were removed, and the plate was washed five times with PBS. The detecting antibody (255-11-biotin) was added to each well (1 µg/mL), and the plate was incubated for another 2 hours. Following washing five times with PBS, 100 μL of 1:1,000 diluted streptavidin-alkaline phosphate conjugate was added to each well and the plate further incubated for 1 hour. The plate was washed again five times with PBS and 100 μL of substrate solution was added to each well and the plate developed until spots became visible. Color development was stopped by washing extensively with tap water and the plate was air dried before spots were counted at Mabtech using an ELISpot reader.

### Peripheral and Intrahepatic Expression of Receptor Pathway Molecules and Immune Cell Markers

Changes in the transcript level of ZBP1/DAI and AIM2 receptor pathway molecules (**Supplementary Table 1**) in blood and liver of woodchucks M8001 and F8003 were determined using real-time PCR assay and woodchuck-specific primers and probes (**Supplementary Table 2**), as described above under whole blood assay and previously (38, 40). A fold-change of ≥2.1 from the pre-treatment baseline was considered a positive result for increased gene expression after *in vivo* treatment with poly(dA:dT).

### Statistical Analysis

Statistical comparisons were performed using unpaired Student’s *t*-test with equal variance for detecting significant changes in PRR pathway activation and WHV replication and secretion in PWHs. *P* values <0.05 were considered significant.

## Results

### Agonistic Stimulation of Viral DNA Sensing Receptors Mediates WHV Suppression

**Figure 1:**
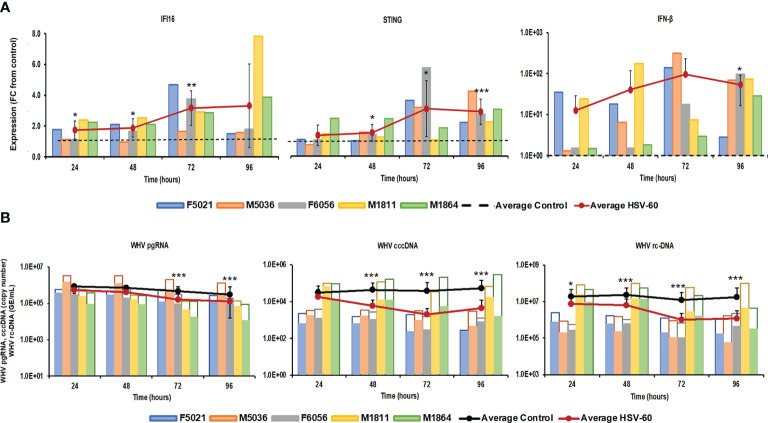
Agonistic activation of IFI16 receptor pathway mediates WHV suppression. PWHs generated from the liver of five woodchucks with CHB were treated with HSV-60 at T0 and again at 48 hours. **(A)** The fold-changes in transcript level of IFI16, STING, and IFN-β over 96 hours are shown, when compared to their transcript level in untreated control PWHs from individual animals at each timepoint, which was set at 1.0 and is indicated by the dotted line. **(B)** The changes in WHV replication (pgRNA and cccDNA) and secretion (rc-DNA) during treatment are presented for untreated control (empty bars) and HSV-60 treated PWHs (colored bars) from individual animals at each timepoint. The average fold-change in gene transcript level or average change in WHV load of control and HSV-60-treated PWHs are presented as solid lines. Horizontal bars represent the standard error of the mean. *P* values representing statistical significance from untreated control PWHs are shown as * for <0.05, ** for <0.01, and *** for <0.001. FC, fold-change.

**Figure 2:**
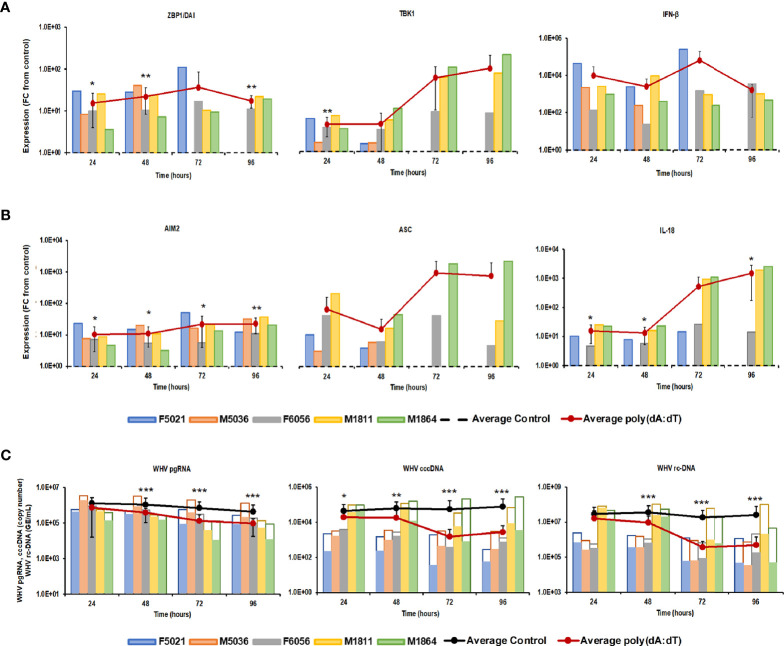
Agonistic activation of ZBP1/DAI and AIM2 receptor pathways mediates WHV suppression. PWHs generated from the liver of five woodchucks with CHB were treated with poly(dA:dT) at T0 and again at 48 hours. The fold-changes in transcript level of **(A)** ZBP1/DAI, TBK1, and IFN-β and **(B)** AIM2, ASC, and IL-18 over 96 hours are shown, when compared to their transcript level in untreated control PWHs from individual animals at each timepoint, which was set at 1.0 and is indicated by the dotted line. **(C)** The changes in WHV replication (pgRNA and cccDNA) and secretion (rc-DNA) during treatment are presented for untreated control (empty bars) and poly(dA:dT)-treated PWHs (colored bars) from individual animals at each timepoint. The average fold-change in gene transcript level or average change in WHV load of control and poly(dA:dT)-treated PWHs are presented as solid lines. Horizontal bars represent the standard error of the mean. *P* values representing statistical significance from untreated control PWHs are shown as * for <0.05, ** for <0.01, and *** for <0.001. FC, fold-change.

Stimulation of viral RNA sensing receptors (i.e., RIG-I, NOD2, TLR3/7/8) using small agonistic molecules has been already explored for the treatment of CHB, as described above, but the antiviral potential of viral DNA sensing receptors, except for TLR9, remains unknown. Agonistic activation of CDSs and inflammasomes within virus-infected hepatocytes, such as IFI16, ZBP1/DAI, and AIM2, could mediate an antiviral effect against HBV *via* the induced effector cytokines, such as IFN-β, IL-1β, and IL-18. For testing these receptors as potential anti-HBV drug targets, PWHs generated from the liver of five woodchucks with CHB were treated with HSV-60 and poly(dA:dT) to stimulate the CDSs IFI16 or ZBP1/DAI and the inflammasome AIM2, respectively. HSV-60 treatment (**Figure 1**) at T0 and again after 48 hours resulted in increased expression of IFI16 receptor, STING adaptor molecule, and IFN-β effector cytokine. Despite the expected variation in individual PWH cultures due to the outbred nature of donor animals, the average peak expression of receptor pathway markers was observed during 72-96 hours (fold-change: IFI16, 3.3; STING, 3.2; IFN-β, 96.0). Receptor pathway upregulation was associated with a pronounced decline in WHV replication (pgRNA and cccDNA) and secretion (rc-DNA). Compared to untreated PWHs obtained from individual animals at each timepoint, the maximum average reduction at 72-96 hours was 0.48 log_10_ for pgRNA, 1.32 log_10_ for cccDNA, and 1.21 log_10_ for rc-DNA. Poly(dA:dT) treatment (**Figure 2**) at T0 and again after 48 hours increased the expression of ZBP1/DAI DNA receptor and AIM2 inflammasome and their respective adaptor molecules TBK1 and ASC and effector cytokines IFN-β and IL-18. The average peak expression for markers of both receptor pathways was observed during 72-96 hours (fold-change: ZBP1/DAI, 36.5; AIM2, 22.3; TBK1, 99.8; ASC, 1,030.8; IFN-β, 64,406.5; IL-18, 1,497.3). Marked declines in WHV replication and secretion were obtained at 72 hours, and the maximum average reduction from untreated PWHs was 0.72 log_10_ for pgRNA, 1.56 log_10_ for cccDNA, and 1.70 log_10_ for rc-DNA. The effect on WHV secretion appeared longer-lasting, as rc-DNA continued to decline and was reduced on average by 1.72 log_10_ at 96 hours. Overall, it appeared that poly(dA:dT) at the selected dose mediated a greater antiviral effect on WHV than HSV-60, most likely due to the activation of two receptors (ZBP1/DAI and AIM2) rather than one receptor (IFI16). Compared to IFI16 receptor stimulation by HSV-60, poly(dA:dT)-mediated activation of the ZBP1/DAI receptor resulted in a 670-fold higher IFN-β expression, in addition to IL-18 expressed by AIM2.

For controlling agonistic PRR activation by HSV-60 and poly(dA:dT) in woodchuck hepatocytes, PWHs were also treated with poly(I:C) and GS-9620 for stimulation of TLR3 (positive control) or TLR7 (negative control), respectively (**Supplementary Figure 1**). This revealed receptor pathway activation and a moderate antiviral effect against WHV by poly(I:C) but not by GS-9620. For confirming the presence of IFI16, ZBP1/DAI, and AIM2 in hepatocytes rather than in immune cells possibly contaminating the PWH cultures, woodchuck WCH-17 hepatoma cells were treated with HSV-60 and poly(dA:dT) (**Supplementary Figure 2**). This demonstrated that all three receptor pathways were inducible in the hepatoma cells, and that the upregulated expression of receptors, adaptor molecules, and effector cytokines after 24 hours, especially of ZBP1/DAI and AIM2, was fairly comparable to those obtained in PWHs.

### Parallel Agonistic Stimulation of Viral DNA Sensing Receptors Enhances WHV Suppression

**Figure 3:**
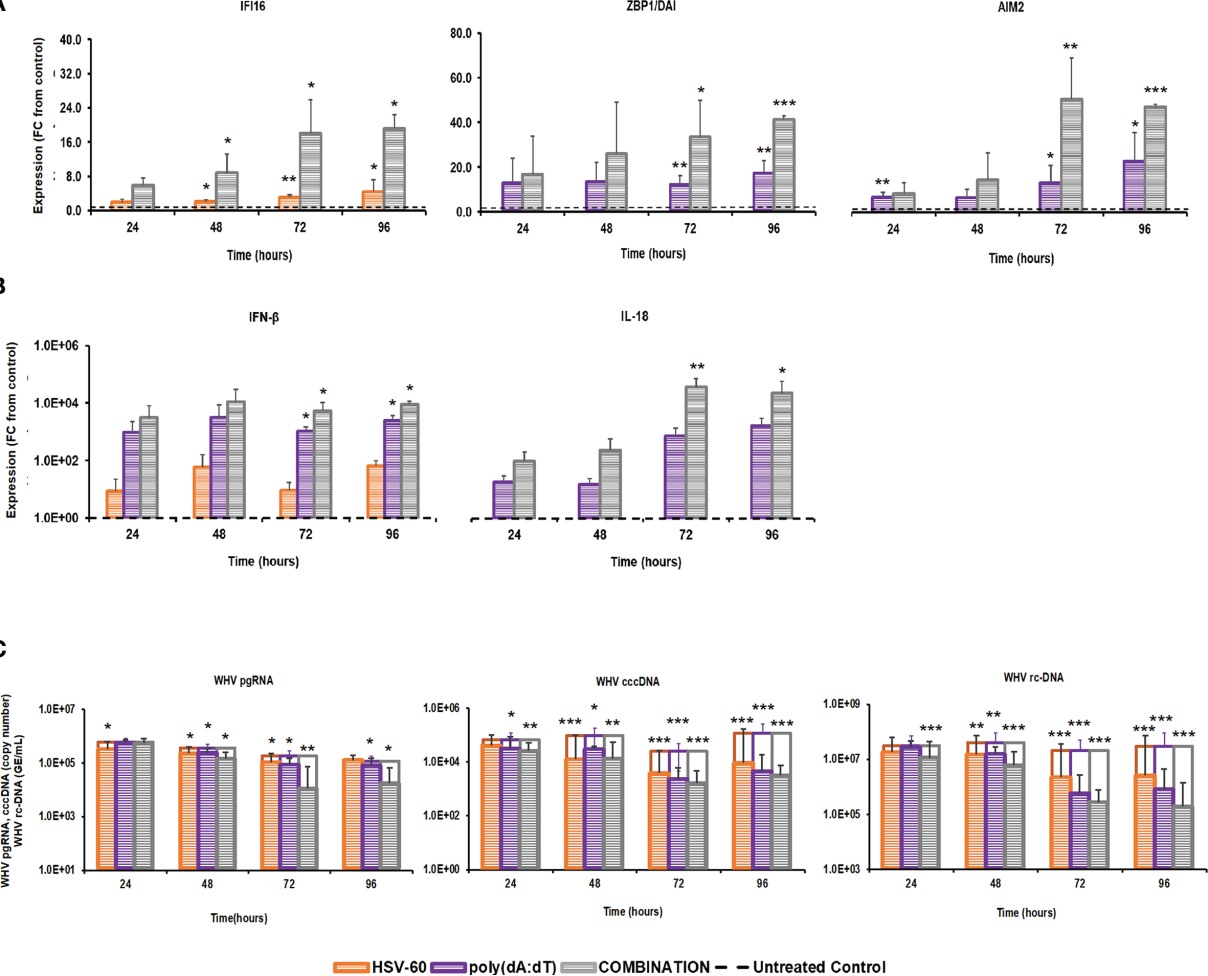
Parallel agonistic activation of IFI16, ZBP1/DAI, and AIM2 receptor pathways enhances WHV suppression. PWHs generated from the liver of three woodchucks with CHB were treated with HSV-60 and poly(dA:dT), alone and in combination, at T0 and again at 48 hours. The fold-changes in transcript level of **(A)** IFI16, ZBP1/DAI, and AIM2 and **(B)** IFN-β and IL-18 over 96 hours are shown, when compared to their transcript level in untreated control PWHs from individual animals at each timepoint, which was set at 1.0 and is indicated by the dotted line. **(C)** The average changes in WHV replication (pgRNA and cccDNA) and secretion (rc-DNA) during mono and combination treatment are presented for untreated control (empty bars) and agonist-treated PWHs (colored bars) at each timepoint. Horizontal bars represent the standard error of the mean. *P* values representing statistical significance from untreated control PWHs are shown as * for <0.05, ** for <0.01, and *** for <0.001. FC, fold-change.

The distinct pathways of the IFI16, ZBP1/DAI, and AIM2 receptors, their production of different effector cytokines, and their location within the cytosol of virus-infected hepatocytes, together with the antiviral effect against WHV mediated by agonistic stimulation, may make them suitable targets for combination treatment. This concept of parallel activation of three DNA receptor pathways was tested in PWHs derived from the liver of three woodchucks with CHB, which were also utilized in the previous monotreatment experiments. Receptor agonism with HSV-60 plus poly(dA:dT) at T0 and again after 48 hours resulted in an average receptor and cytokine expression that was more pronounced than during monotreatment (**Figure 3**). The peak expression of IFI16 (fold-change: HSV-60, mono, 4.5; combo, 19.2) and ZBP1/DAI (fold-change: poly(dA:dT), mono 17.3; combo, 41.4) was observed at 96 hours. AIM2 expression peaked during 72-96 hours (fold-change: poly(dA:dT), mono 22.7; combo, 50.6), while IFN-β expression was maximal during 48-96 hours (fold-change: HSV-60, mono, 65.8; poly(dA:dT), mono, 3,188.5; combo, 10,886.9). Peak IL-18 expression was noted during 72-96 hours (fold-change: poly(dA:dT), mono, 1,497.3; combo, 33,031.0). The maximum antiviral effect of monotreatment and the added antiviral benefit of combination treatment on WHV replication and secretion was observed at 72-96 hours (reduction: pgRNA, HSV-60 mono, 0.31 log_10_; poly(dA:dT) mono, 0.40 log_10_; combo, 1.29 log_10_; cccDNA, HSV-60 mono, 1.00 log_10_; poly(dA:dT) mono, 1.33 log_10_; combo, 1.88 log_10_; rc-DNA, HSV-60 mono, 1.09 log_10_, poly(dA:dT) mono, 1.60 log_10_; combo, 2.22 log_10_). When compared to monotreatment, the increased receptor and cytokine expression during combination treatment correlated with the additional declines in WHV replication and secretion.

### Parallel Agonistic Stimulation of Viral DNA Sensing Receptors Together With Exogenously Added Type-I IFNs Fails to Enhance WHV Suppression

For determining, if the antiviral effect of combination treatment with HSV-60 and poly(dA:dT) is further enhanceable, exogenous type-I IFNs were provided *via* supernatant from woodchuck PBMCs treated with the TLR7 agonist GS-9620. It has been reported that human PBMCs derived from healthy individuals and subsequently treated with GS-9620 produce type-I IFNs and interferon stimulated genes (ISGs), and that treatment of HBV-infected PHHs with this conditioned medium (CM) results in an antiviral effect on viral replication (43). PBMCs obtained from two woodchucks with CHB were treated with GS-9620 for 8 or 24 hours (**Supplementary Figure 3**). At each timepoint, PBMCs were harvested and cell supernatant was collected and combined for subsequent treatment of PWHs with CM (i.e., GS-9620 CM), alone and in combination with HSV-60 and/or poly(dA:dT). Compared to untreated control PBMCs, a maximum expression of IFN-α, IFN-β, and ISG15 in GS-9620 treated PBMCs was observed at eight hours (fold change: M1811, IFN-α, 179.6; IFN-β, 2,098.5; ISG15, 640.3; M1864, IFN-α, 129.8; IFN-β, 87.4; ISG15, 504.7). In PWHs derived from the same two animals and treated with GS-9620 CM at T0 and again after 48 hours (**Supplementary Figure 3**), a maximum reduction of WHV replication and secretion was obtained during 72-96 hours, when compared to untreated control PWHs (reduction: pgRNA, M1811, 0.19 log_10_; M1864, 0.14 log_10_; cccDNA, M1811, 0.11 log_10_; M1864, 0.08 log_10_; rc-DNA, M1811, 0.15 log_10_; M1864, 0.09 log_10_).

**Figure 4:**
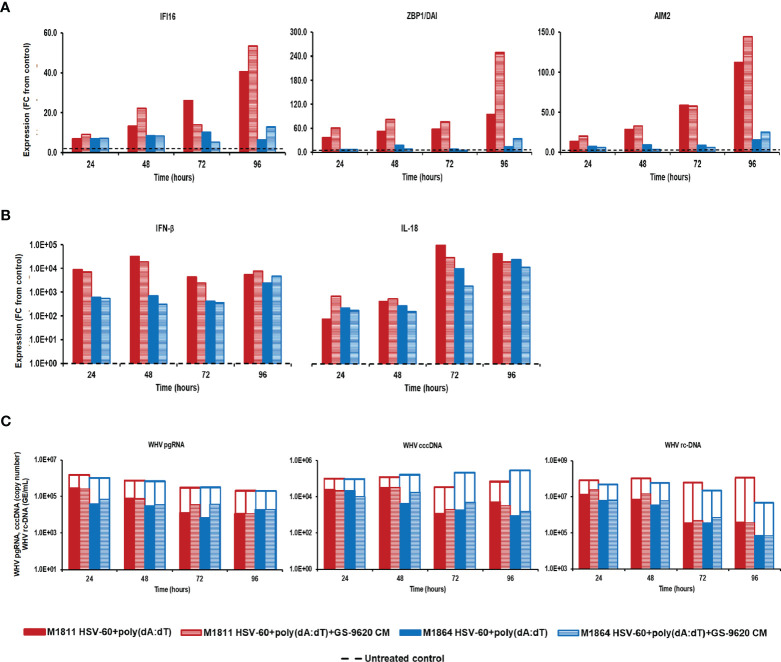
Parallel agonistic activation of IFI16, ZBP1/DAI, and AIM2 receptor pathways together with exogenously added type-I IFNs fails to enhance WHV suppression. PBMCs and PWHs were generated from two woodchucks with CHB, GS-9620 CM was obtained from GS-9620 treated PBMCs, and used to treat PWHs from each animal at T0 and again at 48 hours together with HSV-60 and poly(dA:dT). The fold-changes in transcript level of **(A)** IFI16, ZBP1/DAI, and AIM2 and **(B)** IFN-β and IL18 over 96 hours are shown for treatment with the double (HSV-60 + poly(dA:dT)) or triple combination (HSV-60 + poly(dA:dT) + GS-9620 CM), when compared to their transcript level in untreated control PWHs from individual animals at each timepoint, which that was set at 1.0 and is indicated by the dotted line. **(C)** The changes in WHV replication (pgRNA and cccDNA) and secretion (rc-DNA) during double and triple combination treatment are presented for untreated control (empty bars) and agonist/GS-9620-treated PWHs (colored bars) from individual animals at each timepoints. FC, fold-change.

Treatment of PWHs from the same two animals with the triple combination of HSV-60, poly(dA:dT), and GS-9620 CM at T0 and again after 48 hours induced a higher expression of IFI16, ZBP1/DAI, and AIM2 receptors, when compared to treatment with the double combination of HSV-60 and poly(dA:dT) (**Figure 4**). Peak receptor expression with the double and triple combination was observed at 96 hours in M1811 (fold-change: IFI16, double, 40.5, triple, 53.5; ZBP1/DAI, double, 94.8, triple, 248.7; AIM2, double, 112.1, triple, 144.1) and during 72-96 hours in M1864 (fold-change: IFI16, double, 10.2; triple, 12.8; ZBP1/DAI, double, 13.1, triple, 33.3; AIM2, double, 15.6, triple, 24.8). However, the triple combination failed to increase the expression of IFN-β and IL-18 beyond those obtained with the double combination, except for IFN-β in PWHs of M1864, with a relatively low expression induced by the double combination (fold-change: IFN-β, M1811, double, 31,911.8, triple,19,132.6; M1864, double, 2,375.5, triple, 4,688.3; IL-18, M1811, double, 89,600.0, triple, 27,113.1; M1864, double, 23,149.1, triple, 11,141.0). Contrary, treatment of the same PWHs with HSV-60 and GS-9620 CM resulted in a greater IFI16 receptor and effector cytokine expression, and enhanced the antiviral effect against WHV, when compared to HSV-60 monotreatment (**Supplementary Figure 4**). Treatment of these PWHs with poly(dA:dT) and GS-9620 CM resulted in a mixed expression of ZBP1/DAI and AIM2 receptors, when compared to monotreatment with poly(dA:dT), but was unable to enhance further effector cytokine expression and antiviral effect against WHV (**Supplementary Figure 5**). Thus, based on the cytokine results from triple combination treatment, it further appeared that the additional stimulus by exogenous type-I IFNs was somehow refractory for unknown reasons, since IFN-β and IL-18 expression was reduced during treatment with the triple combination, when compared to the double combination. This was consistent with the observation that the triple combination was unable to enhance the antiviral effect on WHV replication and secretion over that of the double combination in PWHs of M1811 (reduction: pgRNA, double, 1.02 log_10_, triple, 0.89 log_10_; cccDNA, double, 1.46 log_10_, triple, 1.33 log_10_; rc-DNA, double, 2.43 log_10_, triple, 2.49 log_10_) and M1864 (reduction: pgRNA, double, 1.26 log_10_, triple, 0.88 log_10_; cccDNA, double, 2.52 log_10_, triple, 2.27 log_10_; rc-DNA, double, 1.80 log_10_, triple, 1.81 log_10_). The high expression level of effector cytokines already achieved by the double combination could explain the lack of an additional antiviral effect against WHV by the triple combination. Since treatment with exogenous type-I IFNs together with poly(dA:dT) or HSV-60 and poly(dA:dT) was unable to suppress WHV further, this may indicate that the antiviral effect cannot be enhanced indefinitely during short-term treatment once hepatocytes produce and/or secrete effector cytokines at already high (i.e., saturating) levels.

### Poly(dA:dT) Treatment of WHV-Uninfected Woodchucks Stimulates ZBP1 and AIM2 Receptors in the Liver but Also in the Periphery

**Figure 5:**
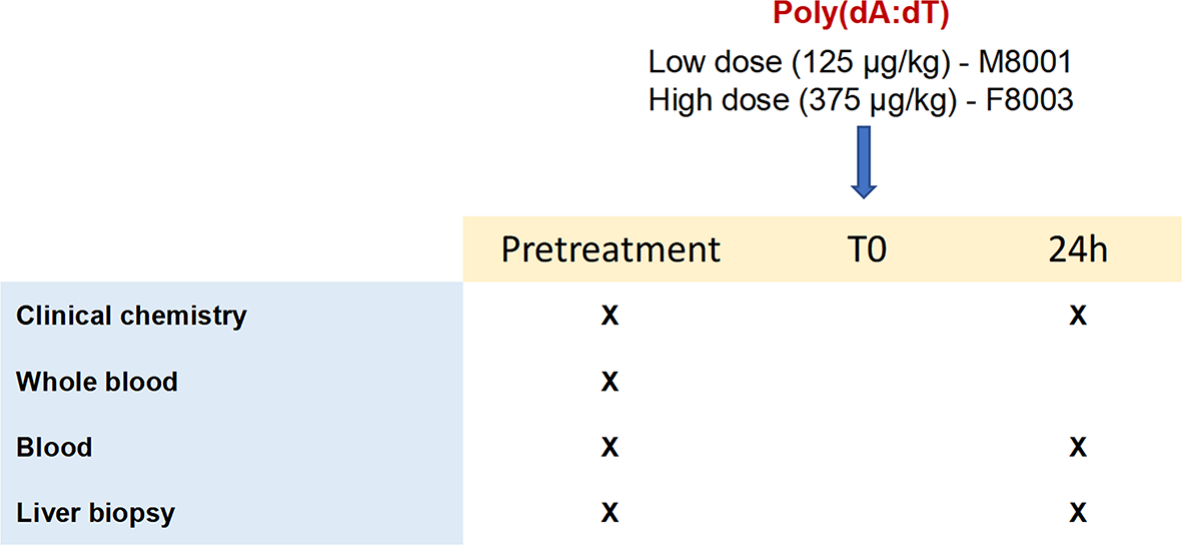
Study design of *in vivo* poly(dA:dT) administration to woodchucks. Poly(dA:dT) mixed in *in vivo-*jetPEI-Gal transfection reagent was administered intravenously as a single dose to two WHV-uninfected adult woodchucks. Animal M8001 received a low dose (125 µg/kg), while animal F8003 received a high dose (375 µg/kg) at T0. Cross marks indicate the time of clinical chemistry measurements and sample collection.

The results from the *in vitro* experiments in PWHs demonstrated that stimulation of more than one PRR by poly(dA:dT) produced a pronounced effect on WHV replication and secretion. For determining the activity and safety of poly(dA:dT) *in vivo*, two WHV-uninfected adult woodchucks were administered a single low (125 μg/kg in M8001) or high (375 μg/kg in F8003) dose of poly(dA:dT) mixed with *in vivo*-jetPEI-Gal transfection reagent (**Figure 5**), and the activation of ZBP1 and AIM2 receptor pathways in blood and liver was determined. The transfection reagent contains a galactose-conjugate for enhanced delivery of nucleic acids to cells expressing galactose-specific membrane lectins, including asialoglycoprotein receptor (ASGP-R) on hepatocytes (42).

**Figure 6:**
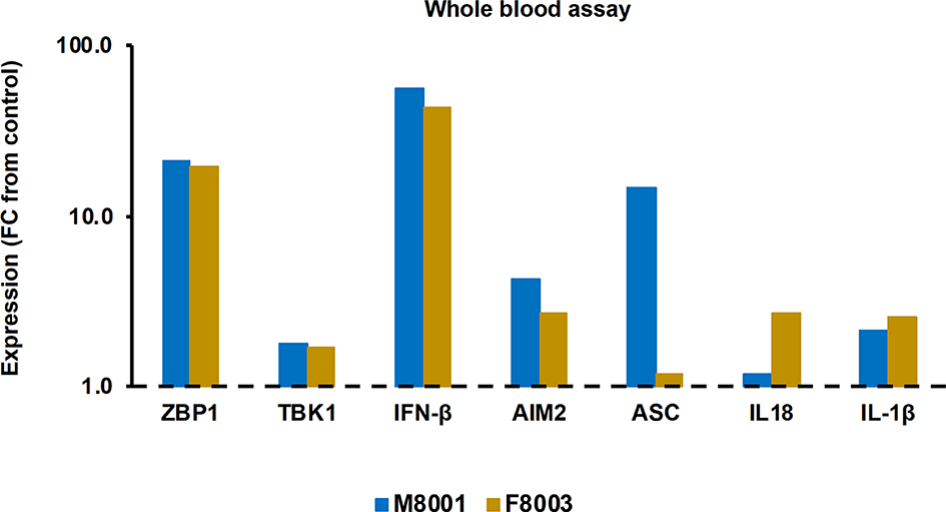
Agonistic activation of ZBP1/DAI and AIM2 receptor pathways in woodchuck blood. Whole blood from animals M8001 and F8003 was treated with poly(dA:dT) in jetOPTIMUS transfection reagent. The fold-changes in transcript level of receptors (ZBP1/DAI and AIM2), adaptor molecules (TBK1 and ASC), and effector cytokines (IFN-β, IL-18, and IL-1β) after 6 hours are shown, when compared to their transcript level in transfection reagent treated control whole blood from individual animals, which was set at 1.0 and is indicated by the dotted line. FC, fold-change.

Prior to administration to woodchucks M8001 and F8003, the jetOPTIMUS transfection reagent with poly(dA:dT) was tested in whole blood from these animals. Whole blood was transfected for 6 hours and blood cells were harvested thereafter. Compared to the transfection control, blood cells of both woodchucks treated with poly(dA:dT) (**Figure 6**) showed increased expression of receptors (fold-change: ZBP1/DAI, M8001, 21.3, F8003, 19.8; AIM2, M8001, 4.4, F8003, 2.7) and effector cytokines, except for IL-18 in M8001 (fold-change: IFN-β, M8001, 56.9, F8003, 44.0; IL-1β, M8001, 2.1, F8003, 2.6; IL-18, M8001, 1.2, F8003, 2.7). The expression of adaptor molecules stayed close to the transfection control baseline, expect for ASC in blood cells of M8001 (fold-change: 14.9).

**Figure 7:**
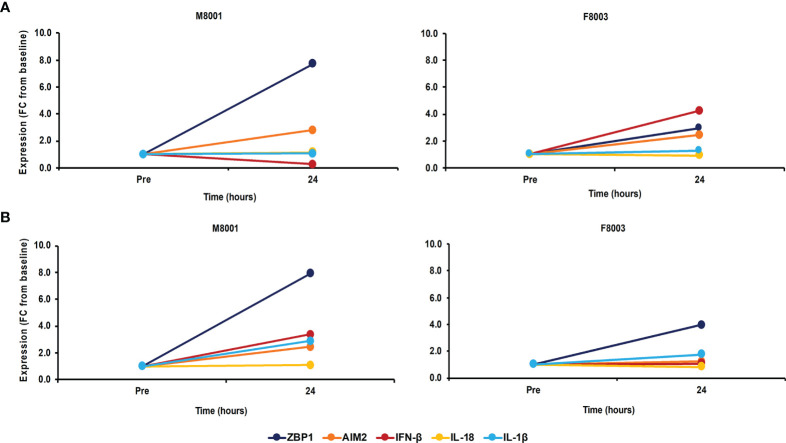
Single dose administration of poly(dA:dT) to woodchucks results in agonistic activation of ZBP1/DAI and AIM2 receptor pathways in the liver and periphery. Liver biopsies and blood from animals M8001 and F8003 were collected 24 hours after intravenous injection of a single low (M8001) or high (F8003) dose of poly(dA:dT) in *in vivo*-jetPEI-Gal transfection reagent. The fold-changes in transcript level of receptors (ZBP1/DAI and AIM2) and effector cytokines (IFN-β, IL-18, and IL-1β) in **(A)** liver and **(B)** blood are shown, when compared to their transcript level at pre-treatment in liver biopsies and blood from the same animals, which was set at 1.0. FC, fold-change.

After the *in vivo* administration of poly(dA:dT) in *in vivo*-jetPEI-Gal transfection reagent to both woodchucks, the intrahepatic activation of receptor pathways was determined in a liver biopsy obtained 24 hours post-treatment (**Figure 7A**). Compared to the liver biopsy obtained prior to treatment, increases in receptor expression were observed in the liver of both animals (fold-change: ZBP1/DAI, M8001 7.7, F8003, 3.0; AIM2, M8001, 2.8, F8003, 2.4), indicating on-target receptor stimulation. However, the expression of most effector cytokines stayed relatively unchanged, expect for IFN-β in F8003 (fold-change: 4.2). The peripheral activation of receptor pathways was further tested in blood obtained 24-hours post-treatment (**Figure 7B**). Compared to blood obtained prior to treatment, increased receptor expression was noted in blood of both animals for ZBP1/DAI (fold-change: M8001, 7.9; F8003, 4.0), but for AIM2 only in M8001 (fold-change: 2.4). The expression of IFN-β and IL-1β was also increased in M8001 (fold-change: IFN-β, 3.4; IL-1β, 2.9), but remained close to the pre-treatment baseline in F8003. No comparable expression changes were observed for IL-18 in both animals.

**Figure 8:**
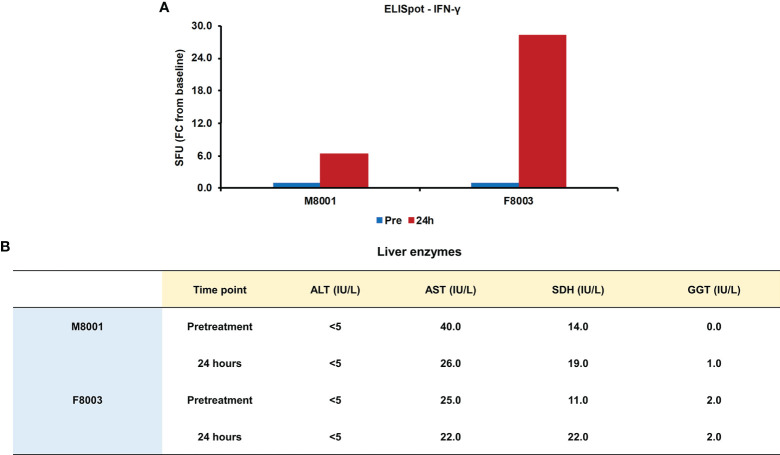
Single dose administration of poly(dA:dT) to woodchucks results in an increased number of IFN-γ secreting blood cells and nearly unchanged liver enzymes. **(A)** PBMCs from animals M8001 and F8003 were isolated 24 hours after intravenous injection of a single low (M8001) or high (F8003) dose of poly(dA:dT) in *in vivo*-jetPEI-Gal transfection reagent and subjected to an ELISpot assay. The fold-changes in spot-forming units (SFU) are shown, when compared to the spot number at pre-treatment in PBMCs from both animals, which was set at 1.0. **(B)** The levels of ALT, AST, SDH, and GGT in serum of both animals measured prior to treatment and at 24 hours post-treatment are shown. The normal range of liver enzyme levels in WHV-uninfected woodchucks is 1-6 IU/mL for ALT, 9-55 IU/mL for AST, and 0.8-2.6 IU/mL for GGT (46). For SDH, the normal range in WHV-uninfected woodchucks is 7-31 IU/mL (47). FC, fold-change; IU, international units.

Since it has been demonstrated that activation of inflammatory pathways can indirectly lead to IFN-γ production by T-cells *via* IL-18 (44, 45), PBMCs obtained from animals M8001 and F8003 24-hours post-treatment were subjected to an ELISpot assay and the number of IFN-γ secreting cells determined (**Figure 8A**). Compared to PBMCs obtained prior to treatment, an increase in IFN-γ secreting cells was observed that appeared dose-dependent (fold-change: M8001, 6.3; F8003, 28.3).

The administration of poly(dA:dT) in *in vivo*-jetPEI-Gal transfection reagent was well-tolerated by both woodchucks, since changes in body weight, body temperature, and hematology were not noted during the 24-hour time course (data no shown). Liver enzymes, including alanine aminotransferase (ALT), aspartate aminotransferase (AST), gamma-glutamyl transferase (GGT), and sorbitol dehydrogenase (SDH), were measured as markers of liver injury prior to treatment and 24 hours post-treatment. In agreement with the normal range of liver enzyme levels in WHV-uninfected woodchucks (46, 47), ALT levels stayed unchanged, AST levels declined somewhat, and only minor but insignificant increases in SDH and GGT levels were observed in both animals or only in one animal, respectively, (**Figure 8B**). Furthermore, when compared to pre-treatment, changes in liver histology associated with agonistic treatment were absent in both animals at 24 hours post-treatment (**Supplementary Figure 6**).

## Discussion

The host RNA and DNA sensing PRRs recognize viral nucleic acids and these PAMPs then trigger the receptor downstream signaling pathways to produce different effector cytokines. These effector cytokines secreted after receptor pathway activation act at the intersection of the innate and adaptive arms of the immunity, and thus influence the direction and magnitude of the adaptive immune response against viral infections (16). Induction of virus-specific adaptive immunity, including T- and B-cell responses, is considered the hallmark for the resolution of either HBV or WHV infection (48, 49). The role of innate immune cells, including natural killer (NK) cells, dendritic cells (DCs), and macrophages, and the involvement of PRRs in the resolution of HBV infection is only just emerging (50, 51). Recent studies demonstrated the *in vitro* recognition of HBV nucleic acids by PRRs, such as cytosolic RIG-I sensing the 5’ ε structure of HBV pgRNA (52) and nucleus resident IFI16 sensing HBV cccDNA (36), thereby promoting the suppression of these key viral molecules. In addition, several viral RNA sensing receptors have been targeted for antiviral effect against CHB *in vivo* (7, 10), but the contribution of other receptors, especially of CDSs and inflammasomes, to HBV resolution and their potential as anti-HBV drug targets are less known. In a recent study, we investigated the previously unknown kinetics of 16 receptors from different families of PRRs in liver and blood of woodchucks during WHV resolution (38). The peak transcripts of these receptors and the maximum protein presence of selected receptors in the liver correlated with the decline in viremia and antigenemia, initially mediated by IFN-γ secreting NK-cells, followed by the responses of virus-specific T- and B-cells (40). Furthermore, a comparison of PRR expression and presence during viral clearance and progression to CHB indicated the involvement of CDSs and inflammasomes in the control of WHV infection during resolution and the lack thereof during persistence (38). These results supported the continued testing of pathway activation and antiviral effects associated with agonistic stimulation of viral DNA sensing receptors in the current study.

Stimulation of the DNA receptors IFI16, ZBP1/DAI, and AIM2 with HSV-60 and poly(dA:dT) agonists resulted in marked IFN-β and IL-18 expression in PWHs from woodchucks with CHB and produced pronounced effects on WHV replication and secretion, indicating that CDS and inflammasome pathways are not actively suppressed or blocked by WHV. Lack of viral interference is further supported by the antiviral effects that are mediated by RNA receptor agonism *in vitro* (TLR3) and *in vivo* (TLR3/7/8/9 or RIG-I), with the latter resulting in reduced or undetectable HBV and WHV viremia and antigenemia levels, and antibody seroconversion in at least subsets of mice and woodchucks (22, 24, 26, 27, 29, 32, 53). Future studies should focus on understanding how HBV/WHV in the setting of CHB avoids efficient detection by PRRs and induction of an innate immune response, both of which is different to the strong receptor pathway activation and antiviral immune response induction during viral resolution. However, the above studies in woodchucks using mRNA expression assays could not differentiate between the PRR location in WHV-uninfected or infected hepatocytes or in non-parenchymal and/or immune cells within the liver, except for a few receptors for which cross-reactive antibodies became available (38). Analyzing immune events in liver at the transcript level mirrors the average from all cells within the biopsy sample, while WHV-infected PWHs could contain minor numbers of non-parenchymal and immune cells. Presence of immune cells in our PWH cultures, such as DCs, could be excluded, as direct treatment with the TLR7 agonist GS-9620 did neither mediate receptor and type-I IFN upregulation nor resulted in an antiviral effect, as also shown for HBV-infected PHHs (43). Cells other than hepatocytes, such as Kupffer cells, present within our PWH cultures may have contributed to the increased IFN-β and IL-18 expression after agonistic activation of IFI16, ZBP1/DAI, and AIM2, because DNA receptor pathways in PHHs are sometimes less activatable by agonists, when compared to human blood-derived macrophages and woodchuck fibroblastoma cells (37, 54). Contrary, another study reported that cGAS levels present in PHHs are sufficient for activation by HBV-derived naked DNA (55). The above studies are in agreement with our previous observation on maximum macrophage accumulation within the liver during WHV resolution in woodchucks (40), which correlated with the peak expression of CDSs and inflammasomes (38). These studies are also in agreement with the response of woodchuck hepatoma cells to agonistic receptor stimulation in the current study. While ZBP1/DAI and AIM2 receptor, adaptor molecule, and cytokine expression was comparable to those in PWHs using poly(dA:dT), IFI16 receptor pathway upregulation with HSV-60 revealed a somewhat lower IFN-β expression in hepatoma cells, suggesting a possible difference between healthy and malignant WHV-infected hepatocytes in regard to the downstream signaling of this receptor.

Mirroring the situation of multiple PRR upregulation in woodchuck liver during WHV resolution for viral control (38), agonistic activation of more than one DNA receptor (i.e., IFI16 *versus* ZBP1/DAI and AIM2), and especially parallel activation of all three receptors, in WHV-infected PWHs increased the overall effector cytokine expression and mediated an antiviral benefit. Furthermore, poly(dA:dT) recognition by RIG-I *via* RNA-polymerase III-transcribed intermediate RNA has been reported (56) and may have contribute to this antiviral benefit. This observation justifies identifying the antiviral potential of HBV/WHV-specific DNA (and RNA) receptors within infected hepatocytes, as the intrinsic immune response induced upon agonistic stimulation will act directly at the site of viral replication. The capability of IFN-α to directly target HBV cccDNA and of type-I IFNs to inhibit HBV pgRNA transcription from cccDNA and to block HBV pgRNA packaging into nucleocapsids have been described previously (57–59). Thus, the induction of type-I IFNs within HBV/WHV-infected hepatocytes by PRR agonism can lead to a marked suppression of viral replication. Furthermore, effector cytokines produced within hepatocytes and then secreted will activate the antiviral functions of innate immune cells that are resident or accumulate in the liver for shaping the adaptive immune response. However, the *in vitro* short-term antiviral effect in WHV-infected PWHs was not enhanceable indefinitely once saturating IFN-β and IL-18 levels were produced by CDSs and inflammasomes and/or secreted, although receptor levels continued to increase. The latter is likely due to a positive feedback loop, since the induction of type-I IFNs can enhance the expression of selected PRRs and ISGs in cell culture and animal models (60). Increased PRR upregulation in the liver was also observed following systemic administration of IFN-α to woodchucks (61). The contribution of the cytokines IFN-α and IL-1β that are also produced by IFI16 and ZBP1/DAI or AIM2 receptors, respectively, to the overall antiviral effect in WHV-infected PWH cultures is unknown, and thus requires additional testing before exploring agonistic stimulation of these receptors for the treatment of CHB.

As a first step in this direction, administration of a single dose of poly(dA:dT) to WHV-uninfected woodchucks was safe, based on unchanged body weight, body temperature, hematology, and clinical chemistry 24 hours later. Furthermore, changes in liver histology were absent and no marked elevation in liver enzymes was observed, except for some minor increases in GGT and SDH in one or both animals, respectively. Following treatment, the intrahepatic expression of ZBP1/DAI and AIM2 receptors and their effector cytokines increased in the liver and periphery of both woodchucks, but the changes did not appear dose-dependent. Consistent with the role of IL-18 as a co-activator of IFN-γ production (44), a dose-dependent increase in T-cells secreting IFN-γ was observed.

The *in vivo*-jetPEI-Gal transfection reagent has been previously applied in mouse models for the liver-targeted delivery of nucleic acids (42), but cross-reactivity to the woodchuck ASGP-R has not been tested. The intrahepatic upregulation of ZBP1/DAI and AIM2 receptors after treatment with poly(dA:dT) suggests that this transfection reagent can be used for targeting woodchuck liver with DNA-based agonists. Since an increased expression of these receptors was also observed in the periphery after treatment, the efficiency of the transfection reagent in liver-specific agonist delivery may be lower than anticipated. Although ASGP-R expression is commonly described to be exclusively on liver cells, low-level expression of this receptor on the surface of activated human PBMCs and T-cells has been described (62, 63), indicating a possible ASPG-R presence on non-hepatic cells. Thus, future woodchuck studies will need to focus on improving the liver-specific transfection efficiency by testing additional transfection reagents, in addition to finding an optimal dose of the poly(dA:dT) agonist. Overall, the present study demonstrated an antiviral benefit that is associated with the agonistic activation of more than one PRR pathway. More specifically, the activation of one cytosolic DNA (i.e., ZBP1/DAI) and one inflammasome (i.e., AIM2) receptor pathway has the advantage of inducing effector cytokines with rather distinct functions that could promote an overall more suitable immune response against chronic HBV infection. The safe *in vivo* administration of poly(dA:dT) that resulted in the intrahepatic upregulation of both receptors further encourages evaluating this agonist for therapeutic efficacy against CHB.

## Data Availability Statement

The original contributions presented in the study are included in the article/**Supplementary Material**. Further inquiries can be directed to the corresponding author.

## Ethics Statement

The animal study was reviewed and approved by the Institutional Animal Care and Use Committee of Georgetown University.

## Author Contributions

MS, BL, XH, KK, MM, SG, and SM contributed to the conception and design of the study. MS, BL, XH, KK, MM, and SM performed the experiments. MS performed the statistical analysis. MS wrote the first draft of the manuscript. All authors contributed to the article and approved the submitted version.

## Funding

MS, MM, SG, and SM were supported in part by grant R01CA166213 of the National Institutes of Health (NIH)/National Cancer Institute (NCI). The funders had no role in the design of the study, in the collection, analyses, or interpretation of data, in the writing of the manuscript, or in the decision to publish the results.

## Conflict of Interest

SM serves occasionally as a paid scientific consultant to Northeastern Wildlife, Inc. (Harris, ID, USA), the only commercial source for woodchucks within the United States, from which the animals of the current study were purchased.

The remaining authors declare that the research was conducted in the absence of any commercial or financial relationships that could be construed as a potential conflict of interest.

## Publisher’s Note

All claims expressed in this article are solely those of the authors and do not necessarily represent those of their affiliated organizations, or those of the publisher, the editors and the reviewers. Any product that may be evaluated in this article, or claim that may be made by its manufacturer, is not guaranteed or endorsed by the publisher.
