# Supplementary figures and images for: Agonistic Activation of Cytosolic DNA Sensing Receptors in Woodchuck Hepatocyte Cultures and Liver for Inducing Antiviral Effects
Source: Front Immunol. 2021 Oct 4;12:745802. doi: 10.3389/fimmu.2021.745802 (PMC8521114; doi:10.3389/fimmu.2021.745802)

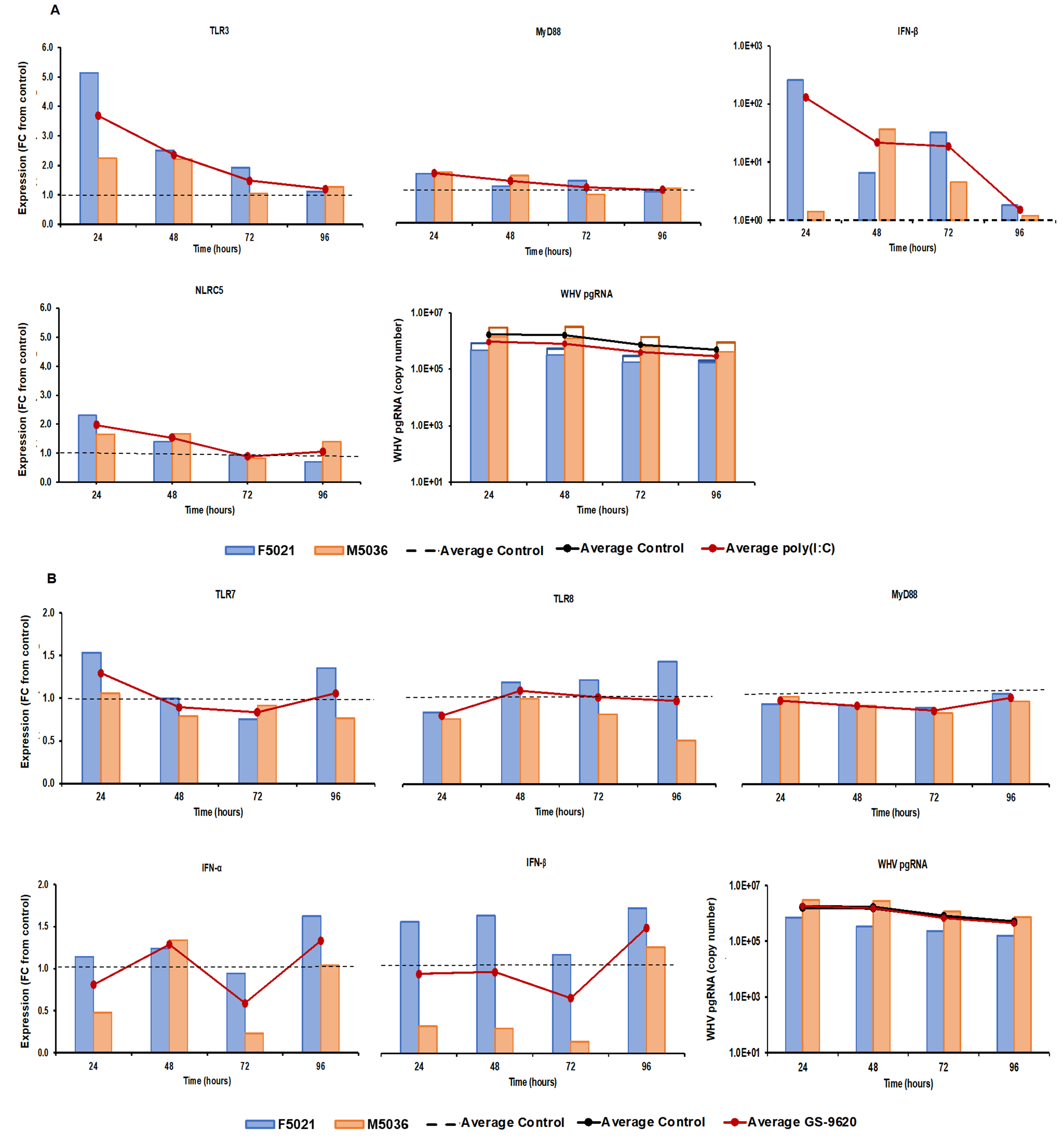

Supplement: Supplementary file 1 [file Image_1.tif]

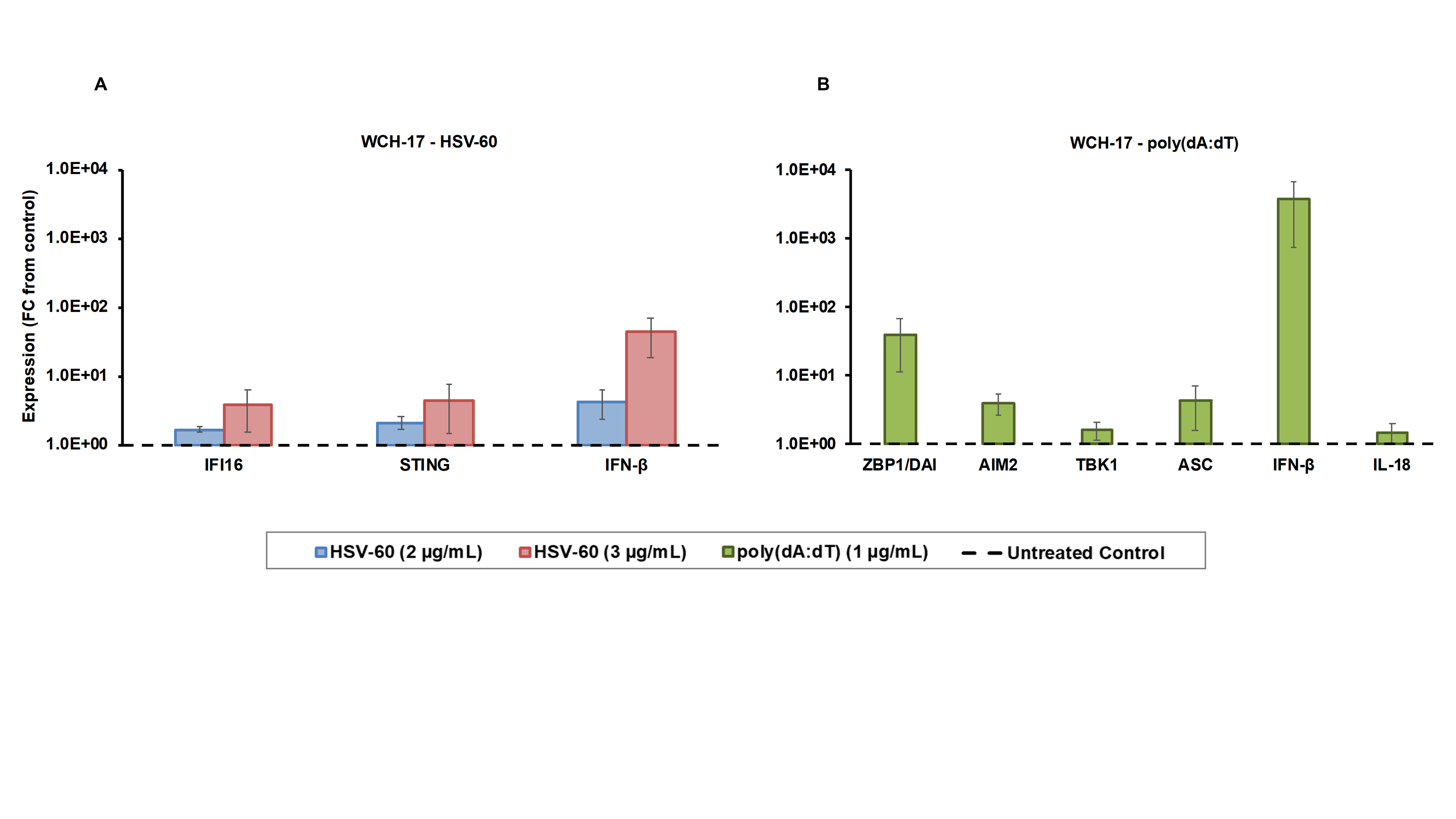

Supplement: Supplementary file 2 [file Image_2.tif]

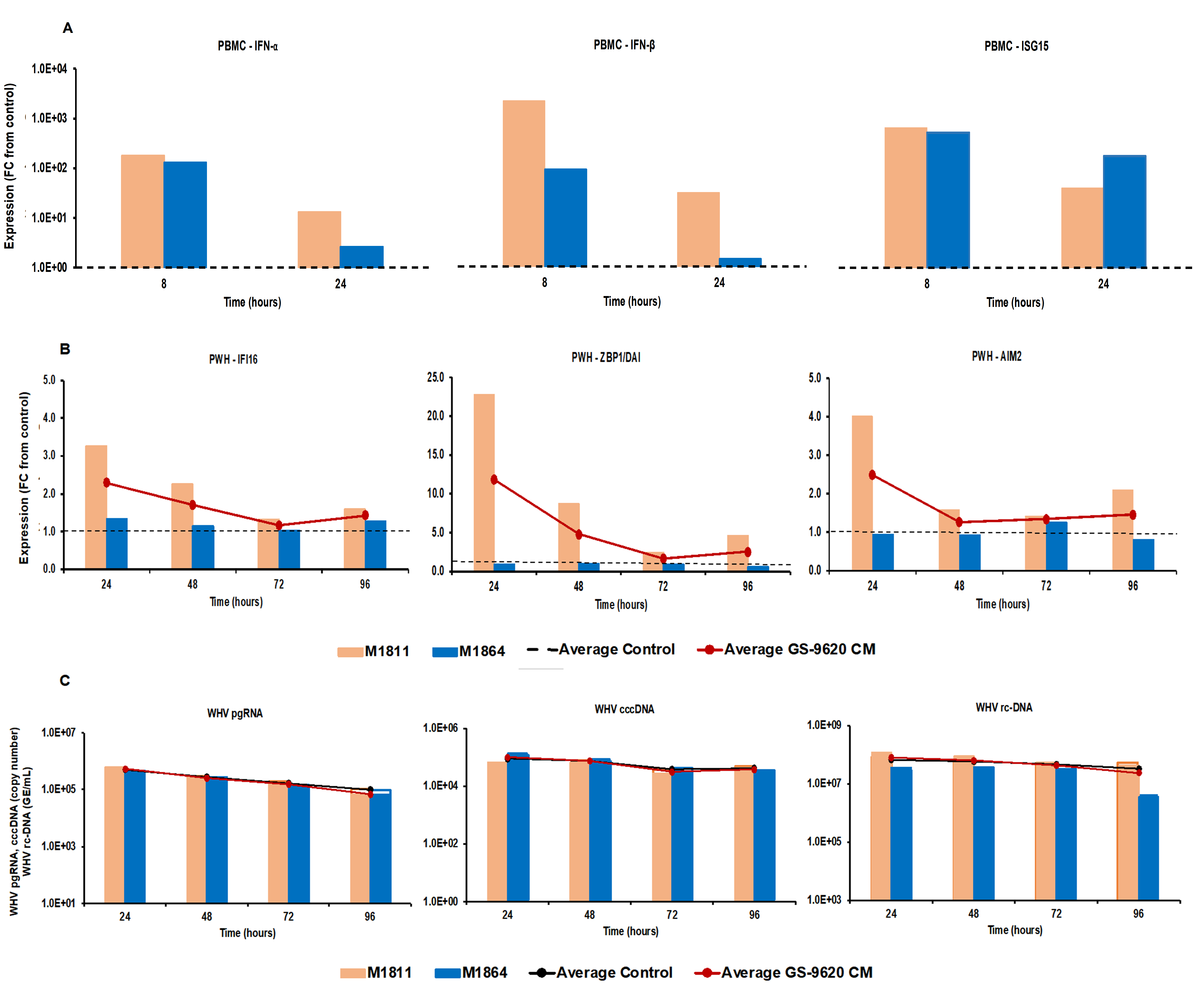

Supplement: Supplementary file 3 [file Image_3.tif]

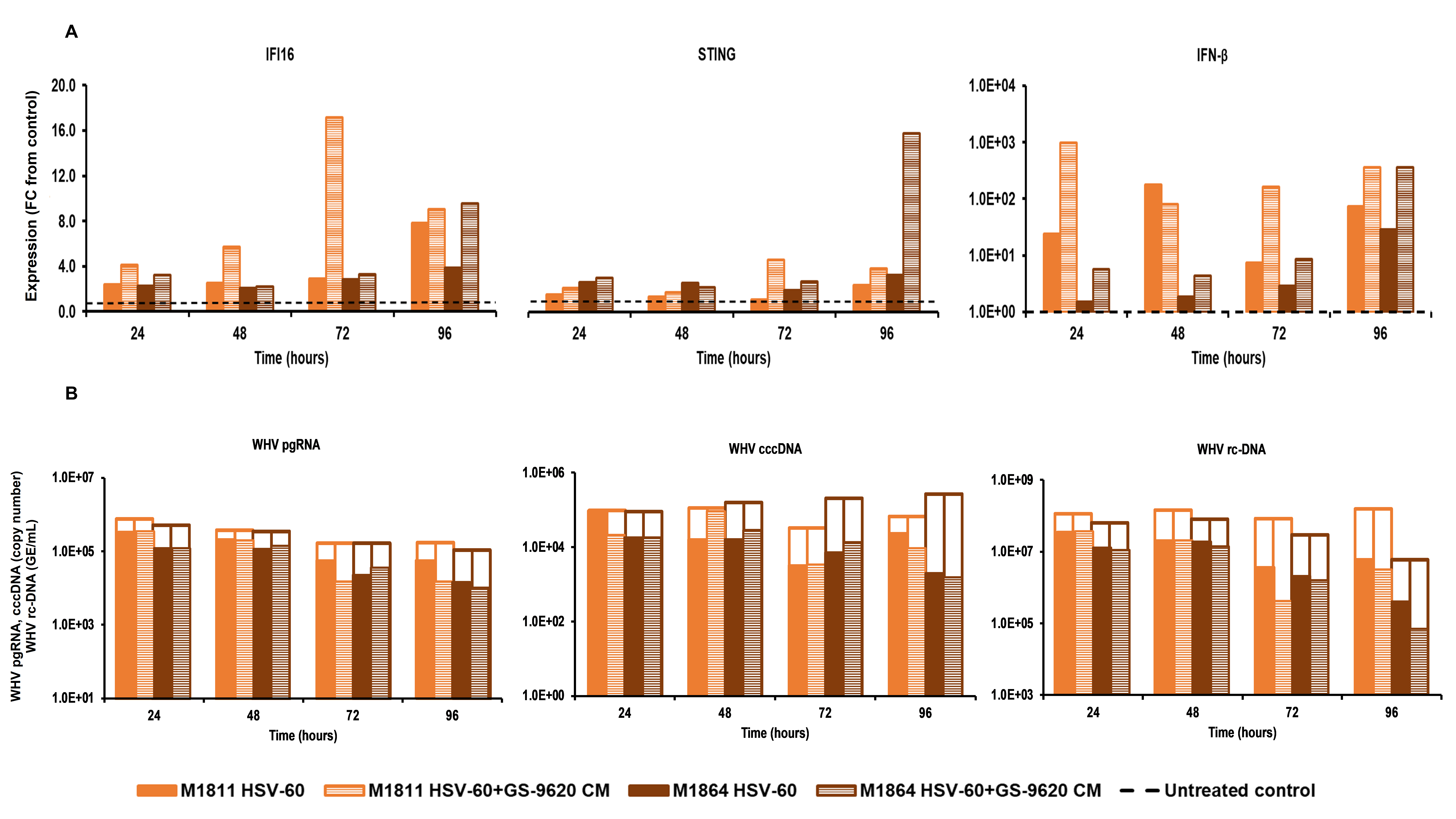

Supplement: Supplementary file 4 [file Image_4.tif]

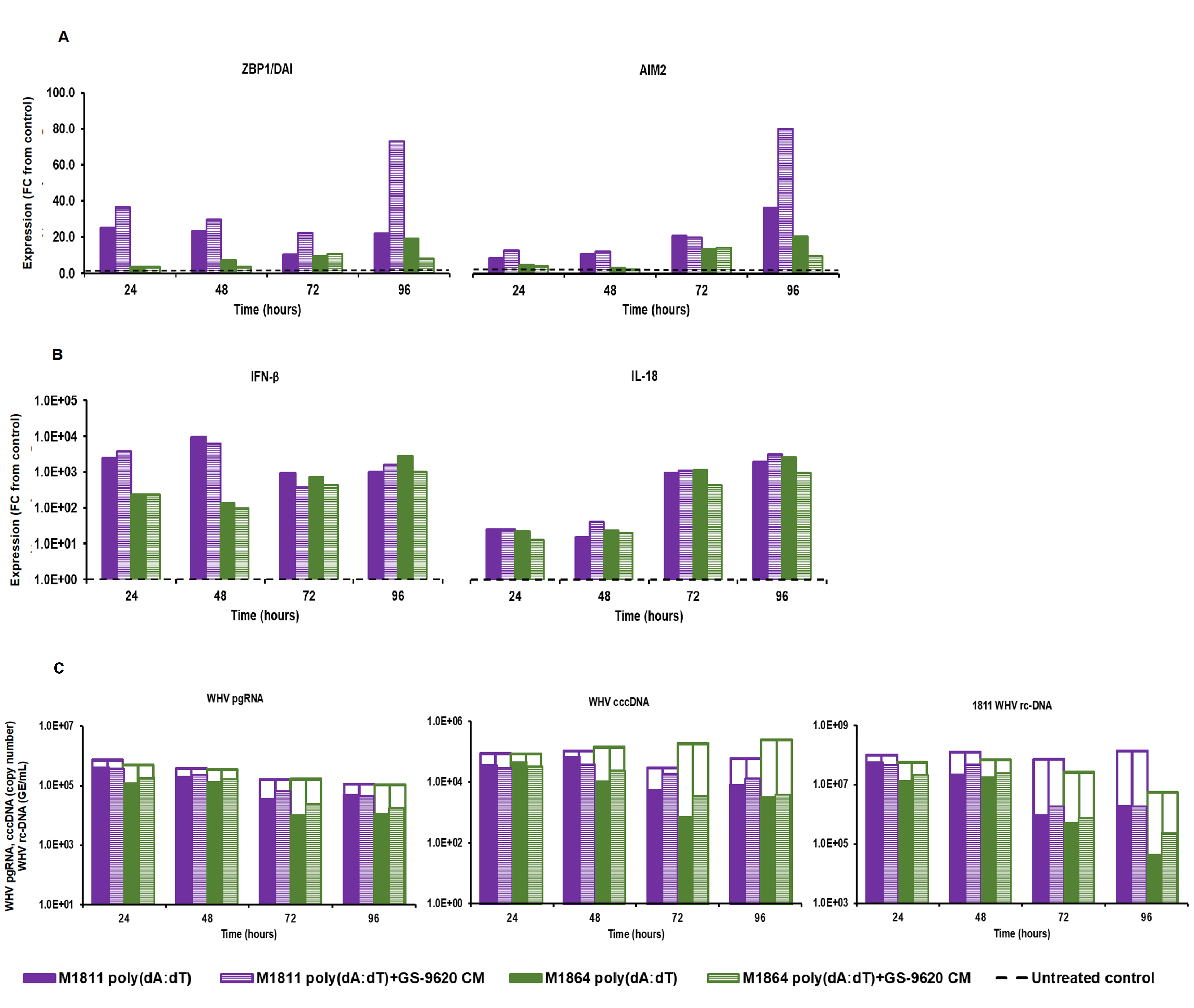

Supplement: Supplementary file 5 [file Image_5.tif]

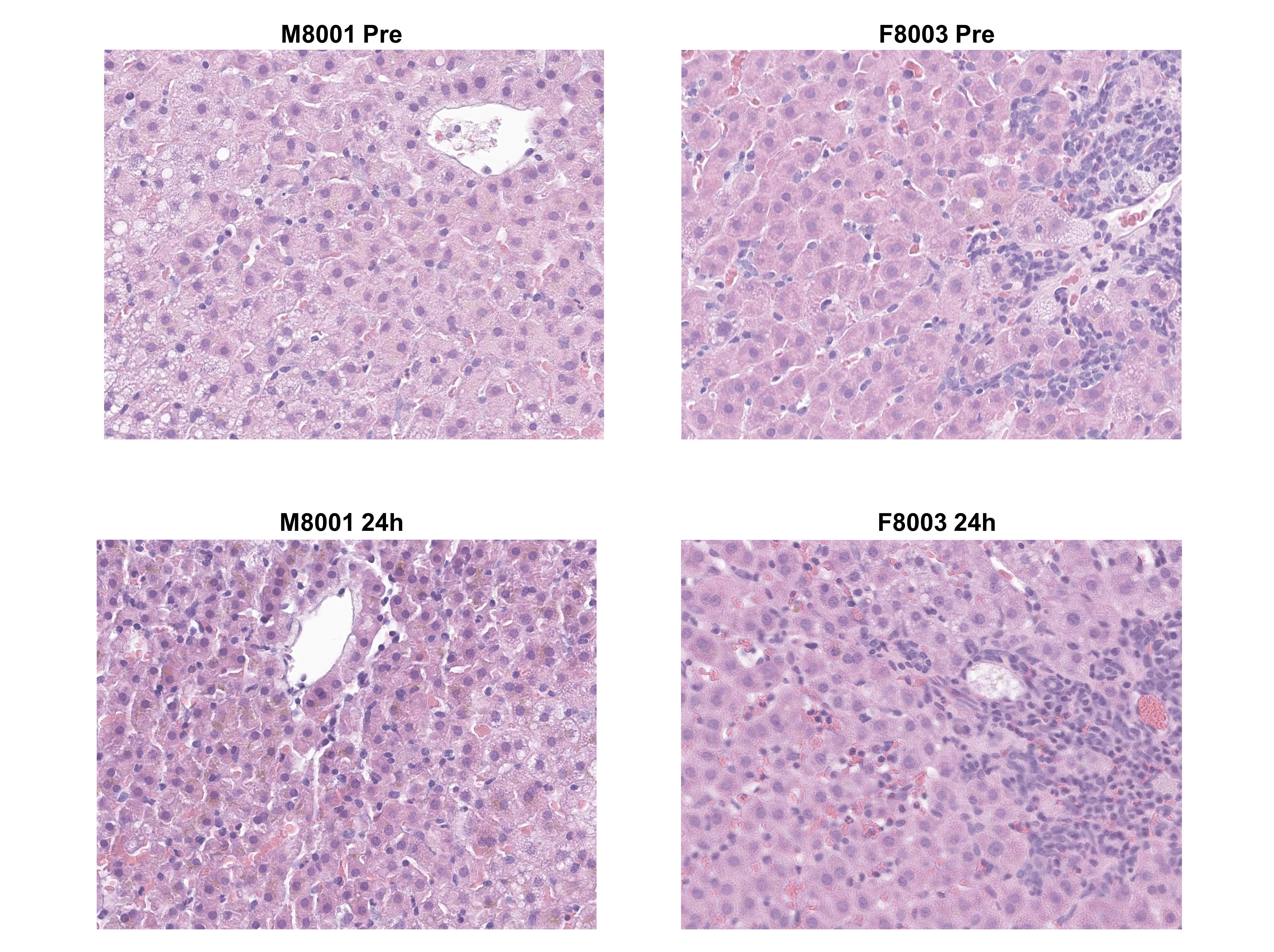

Supplement: Supplementary file 6 [file Image_6.tif]
