# Supplementary material for: Agonistic Activation of Cytosolic DNA Sensing Receptors in Woodchuck Hepatocyte Cultures and Liver for Inducing Antiviral Effects
Source: Front Immunol. 2021 Oct 4;12:745802. doi: 10.3389/fimmu.2021.745802 (PMC8521114; doi:10.3389/fimmu.2021.745802)
Supplement: Supplementary file 7 [file Table_1.docx]

Supplementary Material

# Supplementary Data

**Material and methods**

**Woodchuck hepatocyte isolation and PRR stimulation.**

Primary woodchuck hepatocytes (PWHs) isolated by the collagenase perfusion method (Murreddu et al., 2017) were cultured in 48-well plates at a concentration of 100,000 cells/well for four days at 37^°^C and 5% CO_2_ in complete Williams Media E (WME) medium (Gibco, Gaithersburg, MD), containing 5% fetal bovine serum (FBS; Sigma Aldrich, St. Louis, MO), 10 mM 4-(2-hydroxyethyl)-1-piperazineethanesulfonic acid (HEPES) buffer (Gibco), 100 µg/mL gentamicin (Gibco), 2 µg/mL glucagon (Sigma Aldrich), 1% ITS+1 liquid media supplement (Sigma Aldrich), and 2 mM L-glutamine (Gibco).

Woodchuck WCH-17 hepatoma cells (CRL-2082; ATCC, Manassas, VA) were cultured in 48-well plates at a concentration of 50,000 and 100,000 cells/well for treatment with HSV-60 or poly(dA:dT), respectively, in complete Dulbecco’s modified Eagle medium (DMEM) (Gibco), containing 10% FBS (Sigma Aldrich), 1% sodium pyruvate (Gibco), 1% L-glutamine (Gibco), 1% non-essential amino acids (Gibco), 1% HEPES buffer (Gibco), and 0.5% penicillin/streptomycin (Gibco).

Complete WME medium in PWH cultures was changed every second day during the 4-day period prior to treatment initiation. PWHs and hepatoma cells were treated with agonists, including HSV-60 (2.0 µg/mL for PWHs and 2.0 or 3.0 µg/mL for hepatoma cells), poly(dA:dT) (1.0 µg/mL), poly(I:C) (Invivogen, San Diego, CA; 10.0 µg/mL), and GS-9620 (Gilead Sciences, Foster City, CA) at 1.0 µM as described in (Moreno-Cugnon et al., 2015)) in FBS-free medium using Lipofectamine 3000 transfection reagent (Thermo Fisher Scientific) and OptiMEM (Gibco) by following the manufacturer’s protocol. The transfection reaction lasted for six hours and PWHs and hepatoma cells were subsequently cultured in complete WME or DMEM medium, respectively. PWHs were treated twice during the experiment, initially at T0 and then again after 48 hours. Cell supernatant and hepatocytes were collected every 24 hours over a 96-hour time course. Hepatoma cells were treated only once at T0 and cells were collected 24 hours later. PWH and hepatoma cell cultures treated with Lipofectamine 3000 and OptiMEM medium, but without the agonists, served as an untreated control at each timepoint. For determining agonist-associated cytotoxicity, separate, untreated and treated PWH and hepatoma cell cultures at each timepoint were incubated for one hour with 100 µL of CellTiter-Glo One Solution (Promega, Madison, WI). The cell supernatant was then transferred to 96-well opaque plates (Greiner Bio-One, Monroe, NC), and the number of viable cells assessed *via* a luminescent signal that was proportional to the amount of adenosine triphosphate (ATP) present by using a Centro LB960 luminometer (Berthold Technologies, Bad Wildbad, Germany). No considerable cytotoxicity was observed during the transfection experiments.

**Receptor pathway activation and WHV replication.**

The transcript level of woodchuck immune response genes in PRR agonist-treated PWHs and hepatoma cells was calculated as a fold-change relative to untreated control cells at each timepoint using the formula 2^-Δ^*^Ct^*. WHV rc-DNA was isolated from PWH supernatant using the QIAamp Ultrasens Virus kit (Qiagen, Redwood City, CA) and quantitated by real-time PCR as described previously (Korolowicz et al., 2016). The rc-DNA load was then normalized to the cell number within each PWH culture. Total RNA was isolated from PWHs and hepatoma cells using the RNeasy Mini kit (Qiagen), including on-column digestion with RNase-free DNase I (Qiagen). Total RNA was then subjected to reverse transcription of both WHV pgRNA (only PWHs) and woodchuck immune response gene mRNA (Supplementary Table 1; see below) with the High-Capacity cDNA Reverse Transcription kit (Applied Biosystems, Foster City, CA) using a WHV-specific primer (WHV reverse primer #24; (Freitas et al., 2015)) or random primers, respectively (Murreddu et al., 2017; Suresh et al., 2019). The resulting cDNA was amplified on a 7500 Real Time PCR System or a QuantStudio 3 Real Time PCR System instrument (both Applied Biosystems) using TaqMan or SYBR Green Gene Expression Master mix (both Applied Biosystems) and WHV- (primers #23 and #24, and probe #28; (Freitas et al., 2015)) or woodchuck-specific primers and probes (Supplementary Table 2). 18S rRNA expression was utilized to normalize WHV pgRNA load and target gene expression. The WHV pgRNA load and expression of immune response genes were further normalized to the cell number within each PWH culture. WHV cccDNA was isolated from PWHs and quantified in a TaqMan-based real-time PCR assay with WHV-specific primers (#290 and #291) and probe (#292), as described previously (Freitas et al., 2012; Freitas et al., 2015). The WHV cccDNA load was normalized to the cell number within each PWH culture.

**Peripheral blood mononuclear cell isolation, GS-9620 treatment, and woodchuck immune response gene expression.**

PBMCs were isolated by Ficoll-Paque (Sigma Aldrich) density gradient centrifugation (Menne et al., 2007). PBMCs were cultured in 24-well plates (Corning, Tewksbury, MA) at a concentration of 2.5 million cells/well and treated with 1 µM of GS-9620 in complete AIM-V medium (Thermo Fisher Scientific), as described previously (Moreno-Cugnon et al., 2015) PBMCs were cultured at 37^°^C and 5% CO_2_ and supernatant and cells collected after eight and 24 hours. Medium-treated PBMCs served as an untreated control at each timepoint. Cell supernatant collected at both timepoints was combined and used as a conditioned medium (i.e., GS-9620 CM) for treatment of PWHs. Harvested PBMCs were lysed in RLT buffer (from the QIAmp RNA Blood Mini kit (Qiagen)), containing 1% β-mercaptoethanol (Sigma Aldrich). Total RNA of PBMCs, PWHs, and WCH-17 hepatoma cells was then isolated using the QIAmp RNA Blood Mini kit (Qiagen), including on-column digestion with RNase-free DNase I (Qiagen). Following reverse transcription of woodchuck immune response gene mRNA with the High-Capacity cDNA Reverse Transcription kit (Applied Biosystems) using oligo(dT), cDNA was subjected to real-time PCR, as described above. The transcript level of target genes in the PBMC samples treated with GS-9620 for eight or 24 hours was calculated as a fold-change relative to the untreated control sample at each timepoint using the formula 2^-Δ^*^Ct^*. Treatment of PWHs with GS-9620 CM, alone and in combination with HSV-60 and/or poly(dA:dT), was performed at T0 and again after 48 hours using 30 µL of conditioned medium in a total of 300 µL of complete WME medium. PWHs and cell supernatant were collected, and treatment-associated cytotoxicity was determined every 24 hours over a 96-hour time course, as described above.

**Poly(dA:dT) administration in WHV-uninfected woodchucks.**

The lyophilized poly(dA:dT) (Invivogen) was resuspended in water to obtain a stock solution of 1 mg/mL. The transfection reagent containing poly(dA:dT) was prepared by following the manufacturer’s protocol. In brief, solution A had a total volume of 2 mL and contained 10% glucose in water and poly(dA:dT) at the low (125 µg/kg) or high dose (375 µg/kg). Solution B also had a total volume of 2 mL and contained 10% glucose in water and *in vivo*-jetPEI-Gal transfection reagent (PolyPlus Transfection). Solutions A and B were combined for obtaining a nucleic acid/transfection reagent ratio of 1:6. The combined solution was incubated for 15 minutes at room temperature and then administered to anesthetized woodchucks by intravenous injection.

**Results**

**Agonistic stimulation of viral DNA sensing receptors mediates WHV suppression.**

Treatment with poly(I:C), a widely used TLR3 agonist, served as a positive control for agonistic PRR stimulation by HSV-60 and poly(dA:dT) in woodchuck hepatocytes. PWHs generated from the liver of two animals with CHB were treated with poly(I:C) at T0 and again after 48 hours (Supplementary Figure 1). The average peak expression of TLR3 and IFN-β was observed at 24 hours (fold change: TLR3, 3.7; IFN-β, 130.1). Expression of myeloid differentiation primary response protein 88 (MyD88) adaptor molecule and NOD-like receptor family CARD domain containing 5 (NLRC5), another viral RNA sensing receptor activatable by poly(I:C), remained close to the baseline expression in untreated PWHs. The maximum average reduction in WHV pgRNA upon treatment with poly(I:C) was 0.31 log_10_ at 48 hours, when compared to untreated PWHs. Other WHV replication and secretion markers (i.e., cccDNA and rc-DNA) were not determined in this and the following experiment. Overall, the relatively fast receptor pathway activation within 24 hours after treatment with the first dose of poly(I:C), and the apparent lack of the second dose to further modulate the expression of TLR3, MyD88, and IFN-β, was associated with a moderate antiviral effect. However, the kinetics of receptor pathway activation and WHV suppression by poly(I:C) were different to those obtained with HSV-60 and poly(dA:dT) in PWHs (compare Supplementary Figure 1 with Figures 1 and 2).

Direct treatment of primary human hepatocytes (PHHs) with the TLR7 agonist GS-9620 that activates mainly TLR7, but also TLR8 at high concentrations (Daffis et al., 2020), neither induces ISGs nor mediates an antiviral effect in HBV-infected hepatocytes (Niu et al., 2018). Therefore, as a negative control for agonistic PRR stimulation by HSV-60 and poly(dA:dT) in woodchuck hepatocytes, PWHs from the above two animals were also treated with GS-9620 at T0 and again after 48 hours (Supplementary Figure 1). Changes in the expression of TLR7/8, MyD88, and type-I IFNs remained close to the baseline expression in untreated PWHs, with no apparent antiviral effect on viral pgRNA (maximum average reduction: 0.04 log_10_). Since TLR7/8 are mainly present in antigen presenting cells, such as dendritic cells and B-cells, the absence of receptor pathway activation also indicated rather pure PWH cultures, without contaminating immune cells.

Woodchuck WCH-17 hepatoma cells were treated with agonists for confirming the presence and activatability of IFI16, ZBP1/DAI, and AIM2 receptor pathways in woodchuck hepatocytes. WCH-17 cells received HSV-60 or poly(dA:dT) at T0 in three independent experiments (Supplementary Figure 2). In HSV-60 treated hepatoma cells, the average expression of IFI16, STING, and IFN-β dose-dependently increased after 24 hours (fold-change: IFI16, 1.7-3.8, STING, 2.1-4.5, IFN-β, 4.3-43.2). This was also noted for poly(dA:dT) treated hepatoma cells, in which the average expression of ZBP1/DAI, AIM2, TBK1, ASC, IFN-β, and IL-18 increased after 24 hours (fold-change: ZBP1/DAI, 39.7, AIM2, 4.0, TBK1, 1.6, ASC, 4.4, IFN-β, 3,749.3, IL-18, 1.5). These results indicated that selected viral DNA sensing receptors are present in WHV-infected, malignant hepatocytes and that their downstream pathways are activatable by agonists. The rather comparable expression of receptors, adaptor molecules, and effector cytokines in hepatoma cells and PWHs after 24 hours of treatment (compare Supplementary Figure 2 with Figures 1 and 2) suggested that agonistic treatment of PWHs most likely stimulates viral DNA sensing receptors that are located in WHV-infected, healthy hepatocytes. Of note is that a comparable IFN-β expression in 50,000 hepatoma cells was only obtained with an HSV-60 dose higher than the dose used for treatment of 100,000 PWHs (i.e., 3.0 versus 2.0 µg/mL). This may indicate differential receptor pathway signaling in healthy *versus* malignant hepatocytes.

**Parallel agonistic stimulation of viral DNA sensing receptors together with exogenously added type-I IFNs fails to enhance WHV suppression**

For testing if the high cytokine expression already achieved by combination treatment with HSV-60 and poly(dA:dT) (i.e., the levels of IFN-β and IL-18 produced by hepatocytes and secreted into cell supernatant were already saturating) may be responsible for the lack of an additional antiviral effect by exogenously provided type-I IFNs (i.e., IFN-β) as shown in Figure 4, PWHs from both animals were also treated with the double combination of HSV-60 and GS-9620 CM or poly(dA:dT) and GS-9620 CM. Contrary to treatment with the triple combination of HSV-60, poly(dA:dT), and GS-9620 CM (Figure. 4), treatment with the combination of HSV-60 and GS-9620 CM at T0 and again after 48 hours resulted in a greater IFI16 receptor pathway activation, and also enhanced the antiviral effect against WHV, when compared to HSV-60 monotreatment (Supplementary Figure 4). In PWHs from M1811, peak expression of IFI16 (fold change: HSV-60 mono, 7.8; HSV-60 + GS-9620 CM combo, 17.2) and STING (fold change: mono, 2.3; combo, 4.6) was observed during 72-96 hours, while the maximum IFN-β expression (fold change: mono, 175.2; combo, 969.2) was noted during 24-48 hours. A maximum reduction in WHV replication (pgRNA, mono, 0.38 log_10_; combo, 0.93 log_10_; cccDNA, mono, 1.02 log_10_; combo, 1.00 log_10_), and secretion (rc-DNA, mono, 1.25 log_10_; combo, 2.14 log_10_) was obtained during 72-96 hours. Similarly, in PWHs from M1864, peak expression of IFI16 (fold change: mono, 3.9; combo, 9.6), STING (fold change: mono, 3.2; combo, 15.7), and IFN-β (fold change: mono, 28.5; combo, 361.9) was observed at 96 hours. At the same timepoint, a maximum reduction in WHV replication (pgRNA, mono, 0.78 log_10_; combo, 0.93 log_10_; cccDNA, mono, 2.17 log_10_; combo, 2.27 log_10_), and secretion (rc-DNA, mono, 1.04 log_10_; combo, 1.81 log_10_) were noted. Furthermore, treatment with the combination of HSV-60 and GS-9620 CM enhanced the antiviral effect mediated by monotreatment with GS-9620 CM (Supplementary Figure 4). One limitation of this experiment is that the dose dependent effect of exogenous type-I IFNs in combination with HSV-60 was not tested, for example by increasing the volume of the conditioned medium or by using recombinant IFN beta 1α.

Treatment of PWHs with the combination of poly(dA:dT) and GS-9620 CM also induced a higher expression of ZBP1/DAI and AIM2 receptors (Supplementary Figure 5), when compared to monotreatment with poly(dA:dT), but only in cultures from M1811 (fold-change: ZBP1/DAI, mono, 21.9, combo, 73.0; AIM2, mono, 36.7, combo, 80.5), while cultures from M1864 had a somewhat lower receptor expression (fold-change: ZBP1/DAI, mono, 19.0, combo, 10.8; AIM2, mono, 20.5, combo, 14.4). Similar to treatment with the triple combination of HSV-60, poly(dA:dT), and GS-9620 CM (Figure 4), additional increases in cytokine expression were not observed, except for a slightly higher IL-18 expression in PWHs from M1811 (IFN-β, M1811, mono, 9,403.9, combo, 6,407.3; M1864, mono, 2,811.3, combo, 1,050.9; IL-18, M1811, mono, 1,919.4, combo, 3,047.5; M1864, mono, 2,558.3, combo, 936.4). Consistent with the somewhat reduced cytokine expression, no enhancement of the antiviral effect on WHV replication and secretion was noted (reduction: pgRNA, M1811, mono, 0.54 log_10_, combo, 0.30 log_10_; M1864, mono, 1.11 log_10_, combo, 0.74 log_10_; cccDNA, M1811, mono, 0.91 log_10_, combo, 0.72 log_10_; M1864, mono, 2.44 log_10_, combo, 1.87 log_10_; rc-DNA, M1811, mono, 1.81 log_10_, combo, 1.76 log_10_; M1864, mono, 1.98 log_10_, combo, 1.46 log_10_). As noted before for HSV-60 plus GS-9620 CM, treatment with the combination of poly(dA:dT) and GS-9620 CM also enhanced the antiviral effect mediated by monotreatment with GS-9620 CM (Supplementary Figure 5). Taken together, these results may indicate that treatment with poly(dA:dT) alone induces a saturating level of IFN-β (and IL-18) expression at the used (optimal) dose that is sufficient for inducing a maximal antiviral effect over a 96-hour time course. Contrary, the IFN-β expression level induced by treatment with HSV-60 at the applied (sub-optimal) dose can be increased by adding exogenous type-I IFNs (GS-9620 CM) or by stimulation of additional receptors (i.e., ZBP1/DAI and AIM2) *via* poly(dA:dT) for extra endogenous cytokine production.

**References**

1. Daffis, S., Balsitis, S., Chamberlain, J., Zheng, J., Santos, R., Rowe, W., et al. (2020). Toll-Like Receptor 8 Agonist GS-9688 Induces Sustained Efficacy in the Woodchuck Model of Chronic Hepatitis B. *Hepatology*. doi: 10.1002/hep.31255.
2. Freitas, N., Lukash, T., Rodrigues, L., Litwin, S., Kallakury, B.V., Menne, S., et al. (2015). Infection Patterns Induced in Naive Adult Woodchucks by Virions of Woodchuck Hepatitis Virus Collected during either the Acute or Chronic Phase of Infection. *J Virol* 89(17)**,** 8749-8763. doi: 10.1128/JVI.00984-15.
3. Freitas, N., Salisse, J., Cunha, C., Toshkov, I., Menne, S., and Gudima, S.O. (2012). Hepatitis delta virus infects the cells of hepadnavirus-induced hepatocellular carcinoma in woodchucks. *Hepatology* 56(1)**,** 76-85. doi: 10.1002/hep.25663.
4. Korolowicz, K.E., Iyer, R.P., Czerwinski, S., Suresh, M., Yang, J., Padmanabhan, S., et al. (2016). Antiviral Efficacy and Host Innate Immunity Associated with SB 9200 Treatment in the Woodchuck Model of Chronic Hepatitis B. *PLoS One* 11(8)**,** e0161313. doi: 10.1371/journal.pone.0161313.
5. Menne, S., Tennant, B.C., Gerin, J.L., and Cote, P.J. (2007). Chemoimmunotherapy of chronic hepatitis B virus infection in the woodchuck model overcomes immunologic tolerance and restores T-cell responses to pre-S and S regions of the viral envelope protein. *J Virol* 81(19)**,** 10614-10624. doi: 10.1128/JVI.00691-07.
6. Moreno-Cugnon, L., Esparza-Baquer, A., Larruskain, A., Garcia-Etxebarria, K., Menne, S., Gonzalez-Aseguinolaza, G., et al. (2015). Characterization and genotyping of the DRB1 gene of the major histocompatibility complex (MHC) in the Marmota monax, animal model of hepatitis B. *Mol Immunol* 63(2)**,** 505-512. doi: 10.1016/j.molimm.2014.10.011.
7. Murreddu, M.G., Suresh, M., Gudima, S.O., and Menne, S. (2017). Measurement of Antiviral Effect and Innate Immune Response During Treatment of Primary Woodchuck Hepatocytes. *Methods Mol Biol* 1540**,** 277-294. doi: 10.1007/978-1-4939-6700-1_24.
8. Niu, C., Li, L., Daffis, S., Lucifora, J., Bonnin, M., Maadadi, S., et al. (2018). Toll-like receptor 7 agonist GS-9620 induces prolonged inhibition of HBV via a type I interferon-dependent mechanism. *J Hepatol* 68(5)**,** 922-931. doi: 10.1016/j.jhep.2017.12.007.
9. Suresh, M., Czerwinski, S., Murreddu, M.G., Kallakury, B.V., Ramesh, A., Gudima, S.O., et al. (2019). Innate and adaptive immunity associated with resolution of acute woodchuck hepatitis virus infection in adult woodchucks. *PLoS Pathog* 15(12)**,** e1008248. doi: 10.1371/journal.ppat.1008248.
