# Supplementary material for: Agonistic Activation of Cytosolic DNA Sensing Receptors in Woodchuck Hepatocyte Cultures and Liver for Inducing Antiviral Effects
Source: Front Immunol. 2021 Oct 4;12:745802. doi: 10.3389/fimmu.2021.745802 (PMC8521114; doi:10.3389/fimmu.2021.745802)
Supplement: Supplementary file 8 [file Table_2.docx]

Supplementary Material

**Supplementary Table 1. PRRs and their downstream adaptor and effector molecules analyzed in woodchuck cells.**

| Marker Group | Genes |
| --- | --- |
| Pattern Recognition Receptors | NLRC5 = NOD-like receptor family CARD domain containing 5 |
|  | TLR3 = Toll-like receptor 3 |
|  | TLR7 = Toll-like receptor 7 |
|  | TLR8 = Toll-like receptor 8 |
|  | ZBP1/DAI = Z-DNA-binding protein 1 or DNA-dependent activator of interferon regulatory factors |
|  | IFI16 = Interferon-gamma inducible protein 16 |
|  | AIM2 = Absent in melanoma 2 |
| Adaptor molecules | MyD88 = Myeloid differentiation primary response protein 88 |
|  | MAVS = Mitochondrial antiviral signaling protein |
|  | STING = Stimulator of interferon genes |
|  | TBK1 = TANK-binding kinase 1 |
|  | ASC = Apoptosis-associated spike-like protein coding CARD |
| Cytokines | IFN-α = Interferon-alpha |
|  | IFN-β = Interferon-beta |
|  | IL-1β = Interleukin-1beta |
|  | IL-18 = Interleukin-18 |
| Interferon-stimulated genes | ISG15 = Interferon-stimulated gene 15 |
