# Supplementary material for: Agonistic Activation of Cytosolic DNA Sensing Receptors in Woodchuck Hepatocyte Cultures and Liver for Inducing Antiviral Effects
Source: Front Immunol. 2021 Oct 4;12:745802. doi: 10.3389/fimmu.2021.745802 (PMC8521114; doi:10.3389/fimmu.2021.745802)
Supplement: Supplementary file 9 [file Table_3.docx]

Supplementary Material

**Supplementary Table 2. Oligonucleotides used for analysis of PRRs and their downstream adaptor and effector molecules in woodchuck cells.**

| Gene | Primers and Probe | Sequence |
| --- | --- | --- |
| NLRC5 | F | 5’- ATGTCTGTGCCTCACCAATCATGT -3’ |
|  | R | 5’- ATGTGGCACTTACATGGGACTTGT -3’ |
|  | P | 5’- ACCCAGGCCATTGACATGGGACACAT -3’ |
| TLR3 | F | 5’- GACCAGGTGTCTCTAAAGTTCC -3’ |
|  | R | 5’- CAGGTACAATCAAACGGGTTAAAG -3’ |
|  | P | 5’- CAGTTGAAAGAATGTGTTTGGGCCAGT -3’ |
| TLR7 | F | 5’- GGCACATTCTGAAGAGAGTTACTG -3’ |
|  | R | 5’- TGATCTAAGTGGAAATTGCCCTCG -3’ |
|  | P | 5’- TGTTCCATTTCCTTGCACACCTTGTGA -3’ |
| TLR8 | F | 5’- CAATGGCAATGGCCAAGTATTTA -3’ |
|  | R | 5’- CAGTTCACGGAGAGCATTGT -3’ |
|  | P | 5’ - TCCAAGTATTTGACCTGAGGCACAGC -3’ |
| ZBP1/DAI | F | 5’- CCCAGATGTGTAGATGAGGAAC -3’ |
|  | R | 5’- AAACAGGGACGTAGCCAAA -3’ |
|  | P | 5’- CCCAGTGTGAATCCTCCCATGTCA -3’ |
| IFI16 | F | 5’- CCGAAGATCCATCAGGCAAA -3’ |
|  | R | 5’- CTACTGGATGATGCTGGAGATG -3’ |
|  | P | 5’- TCATCCAGAGGAATCCAGGGTCCT -3’ |
| AIM2 | F | 5’- CACTGATGAGGAACTGGATAGG -3’ |
|  | R | 5’- TGGCTAACTCTGTCCTGTTTG -3’ |
|  | P | 5’- CACGATTGCCAAGAGCAAACTGCA -3’ |
| MyD88 | F | 5’- CATCTTCCACCTCACTTTCTCTAC -3’ |
|  | R | 5’- CCAAGAGAGCCAGAGCAATAC -3’ |
|  | P | 5’- AGCTGTGTTCAAACCACT -3’ |
| STING | F | 5’- CTATGAGCTTCTGGAGAATGGG -3’ |
|  | R | 5’- CTCTGCCATCCTGTGACATG -3’ |
|  | P | 5’- CTGTGTCCTGGAGTATGCCACCC -3’ |
| TBK1 | F | 5’- CTTATCTATGAAGGACGACGCTTA -3’ |
|  | R | 5’- CCCGGCTTACAACGAAGATAG -3’ |
| ASC | F | 5’- GGATATGACCACATTCGTAGGG -3’ |
|  | R | 5’- GGTCACAGTCTTCGAGTATCTT -3’ |
| IFN-α | F | 5’- CTCAAGCTGTTGCTGTCCTC -3’ |
|  | R | 5’- CTTCTGGGTGCTGAAGAGGT -3’ |
|  | P | 5’- CCAGATGACCCAGCAGATCCTCA -3’ |
| IFN-β | F | 5’- GAATGAAAGGCCTGCAGAGT -3’ |
|  | R | 5’- GGATGTTTGATCTTCTTGGG -3’ |
|  | P | 5’- CTTGAAGTCCATCCTGTCACTGAGGC -3’ |
| IL-1β | F | 5’- GACCGAATCTGAGGCAACAA -3’ |
|  | R | 5’- GGGAAGGAAGAGAAGACAAGAG -3’ |
|  | P | 5’- TCCATGAGCTTTGTGCTGGGAGAA -3’ |
| IL-18 | F | 5’- CCTGTCCTTCTGGTTTGTATG -3’ |
|  | R | 5’- GGGAAGGAAGAGAAGACAAGAG -3’ |
| ISG15 | F | 5’- CTGTTCTGGCTGAGCTTCG -3’ |
|  | R | 5’- GCAGGTTCAGAAACACAGTGC -3’ |
|  | P | 5’- GGGAGTATGGACTCACCCCT -3’ |
| 18S rRNA | F | 5’- GTAACCCGTTGAACCCCATT -3’ |
|  | R | 5’- GGGACTTAATCAACGCAAGC -3’ |
|  | P | 5’- GCAATTATTCCCCATGAACG -3’ |

F: forward primer; R: reverse primer; P: probe.
