# Supplementary material for: Agonistic Activation of Cytosolic DNA Sensing Receptors in Woodchuck Hepatocyte Cultures and Liver for Inducing Antiviral Effects
Source: Front Immunol. 2021 Oct 4;12:745802. doi: 10.3389/fimmu.2021.745802 (PMC8521114; doi:10.3389/fimmu.2021.745802)
Supplement: Supplementary file 10 [file Table_4.docx]

Supplementary Material

# Supplementary Figure Legends

**Supplementary Figure 1. Positive and negative controls of agonistic PRR pathway activation.** PWHs generated from the liver of two woodchucks with CHB were treated with (**A**) poly(I:C) as a positive control and (**B**) GS-9620 as a negative control at T0 and again at 48 hours. The fold-changes in transcript level of (**A**) TLR3, MyD88, IFN-β, and NLRC5 and (**B**) TLR7, TLR8, MyD88, IFN-α, and IFN-β over 96 hours are shown, when compared to their transcript level in untreated control PWHs from individual animals at each timepoint, which was set at 1.0 and is indicated by the dotted line. The changes in WHV replication (pgRNA) during treatment are presented for untreated control (empty bars) and agonist-treated PWHs (colored bars) from individual animals at each timepoint. The average fold-change in gene transcript level or average change in WHV load are presented as solid lines. Abbreviations: FC, fold-change.

**Supplementary Figure 2. Agonistic activation of IFI16 or ZBP1/DAI and AIM2 receptor pathways in woodchuck hepatoma cells.** WCH-17 cells were treated in three independent experiments with **(A)** HSV-60 at two separate doses or **(B)** poly(dA:dT) at one dose at T0. The average fold-changes in transcript level of **(A)** IFI16, STING, and IFN-β and **(B)** ZBP1/DAI, AIM2, TBK1, ASC, IFN-β, and IL-18 after 24 hours are shown, when compared to their transcript level in untreated control hepatoma cells, which was set at 1.0 and is indicated by the dotted line. Horizontal bars represent the standard error of the mean. Abbreviation: FC, fold-change.

**Supplementary Figure 3. Type-I IFN and ISG induction in PBMCs by GS-9620 and IFI16, ZBP1/DAI, and AIM2 receptor stimulation and WHV suppression in PWHs by supernatant from GS-9620 treated PBMCs.** PBMCs and PWHs were generated from blood and liver of two woodchucks with CHB. PBMCs were treated with GS-9620 for 8 or 24 hours and the supernantant was collected at each timepoint and combined. This conditioned medium (GS-9620 CM) was then used to treat PWHs from each animal at T0 and again at 48 hours. The fold-changes in transcript level of **(A)** IFN-α, IFN-β, and ISG15 in PBMCs after 8 and 24 hours of GS-9620 treatment and **(B)** IFI16, ZBP1/DAI, and AIM2 in PWHs over 96 hours during GS-9620 CM treatment are shown, when compared to their transcript level in untreated control PBMCs or PWHs from individual animals at each timepoint, which was set at 1.0 and is indicated by the dotted line. **(C)** The changes in WHV replication (pgRNA and cccDNA) and secretion (rc-DNA) during monotreatment are presented for untreated control (empty bars) and GS-9620 CM-treated PWHs (colored bars) from individual animals at each timepoint. The average fold-change in gene transcript level or average change in WHV load are presented as solid lines in (B) and (C). Abbreviations: FC, fold-change.

**Supplementary Figure 4. Agonistic activation of the IFI16 receptor pathway together with exogenously added type-I IFNs enhances WHV suppression.** PBMCs and PWHs were generated from two woodchucks with CHB, GS-9620 CM was obtained as described in the legend to Supplementary Figure 3, and was used to treat PWHs from each animal at T0 and again at 48 hours together with HSV-60. (**A**) The fold-changes in transcript level of IFI16, STING, and IFN-β over 96 hours are shown for monotreatment (HSV-60) or combination treatment (HSV-60 + GS-9620 CM), when compared to their transcript level in untreated control PWHs from individual animals at each timepoint, which was set at 1.0 and is indicated by the dotted line. (**B**) The changes in WHV replication (pgRNA and cccDNA) and secretion (rc-DNA) during mono and combination treatment are presented for untreated control (empty bars) and agonist/GS-9620 CM-treated PWHs (colored bars) from individual animals at each timepoint. Abbreviations: FC, fold-change.

**Supplementary Figure 5. Agonistic activation of ZBP1/DAI and AIM2 receptor pathways together with exogenously added type-I IFNs fails to enhance WHV suppression.** PBMCs and PWHs were generated from two woodchucks with CHB, GS-9620 CM was obtained as described in the legend to Supplementary Figure 3, and was used to treat PWHs from each animal at T0 and again at 48 hours together with poly(dA:dT). The fold-changes in transcript level of **(A)** ZBP1/DAI and AIM2 and **(B)** IFN-β and IL18 over 96 hours are shown for monotreatment (poly(dA:dT) or combination treatment (poly(dA:dT) + GS-9620 CM), when compared to their transcript level in untreated control PWHs from individual animals at each timepoint, which was set at 1.0 and is indicated by the dotted line. **(C)** The changes in WHV replication (pgRNA and cccDNA) and secretion (rc-DNA) during mono and combination treatment are presented for untreated control (empty bars) and agonist/GS-9620 CM-treated PWHs (colored bars) from individual animals at each timepoint. Abbreviations: FC, fold-change.

**Supplementary Figure 6. Single dose administration of poly(dA:dT) to woodchucks is not associated with changes in liver histology.** Liver biopsies from animals M8001 and F8003 were collected 24 hours after intravenous injection of a single low (M8001) or high (F8003) dose of poly(dA:dT) in *in vivo*-jetPEI-Gal transfection reagent. Images of hematoxylin and eosin (H&E) stained liver tissues obtained at pre-treatment (top panel) and at 24 hours post-treatment (bottom panel) are shown for both animals.
